# Transitional Care Interventions for Older Residents of Long-term Care Facilities: A Systematic Review and Meta-analysis

**DOI:** 10.1001/jamanetworkopen.2022.10192

**Published:** 2022-05-04

**Authors:** Kelly Birtwell, Claire Planner, Alexander Hodkinson, Alex Hall, Sally Giles, Stephen Campbell, Natasha Tyler, Maria Panagioti, Gavin Daker-White

**Affiliations:** 1 National Institute for Health Research, School for Primary Care Research, Manchester Academic Health Science Centre, University of Manchester, Manchester, United Kingdom; 2 NIHR Greater Manchester Patient Safety Translational Research Centre, University of Manchester, Manchester, United Kingdom; 3 Division of Nursing, Midwifery and Social Work, School of Health Sciences, Faculty of Biology, Medicine and Health, University of Manchester, Manchester, United Kingdom

## Abstract

**Importance:**

Residents of long-term care facilities (LTCFs) experience high hospitalization rates, yet little is known about the effects of transitional care interventions for these residents.

**Objective:**

To assess the association of transitional care interventions with readmission rates and other outcomes for residents of LTCFs who are 65 years and older and LTCF staff and to explore factors that potentially mitigate the association.

**Data Sources:**

MEDLINE, EMBASE, PsycINFO, Cochrane Central Register of Controlled Trials, and Cumulative Index to Nursing and Allied Health Literature were searched for English-language studies published until July 21, 2021. Associated qualitative studies were identified using aspects of the CLUSTER (citations, lead authors, unpublished materials, searched Google Scholar, tracked theories, ancestry search for early examples, and follow-up of related projects) methodology.

**Study Selection:**

Controlled design studies evaluating transitional care interventions for residents of LTCFs 65 years and older were included. Records were independently screened by 2 reviewers; disagreements were resolved through discussion and involvement of a third reviewer. From 14 538 records identified, 15 quantitative and 4 qualitative studies met the eligibility criteria.

**Data Extraction and Synthesis:**

The study followed the Preferred Reporting Items for Systematic Reviews and Meta-analyses (PRISMA) guidelines. Data were extracted by one reviewer and checked by a second reviewer. Fixed-effect and random-effects models were used according to the number of studies reporting the outcomes of interest.

**Main Outcomes and Measures:**

The primary outcome consisted of 30-, 60-, and 90-day readmission rates (hospital and emergency department [ED]). Other outcomes included length of stay, functional independence (Barthel score), and quality of life. The *I*^2^ statistic was used to quantify heterogeneity.

**Results:**

Of 14 538 records identified from searches, 15 quantitative studies (totaling 32 722 participants or records) and 4 qualitative studies were included. People allocated to transitional care interventions were 1.7 times less likely to be readmitted to the hospital or ED compared with those in control groups (14 studies; odds ratio, 1.66 [95% CI, 1.18-2.35]; *I*^2^ = 81% [95% CI, 70%-88%]). Length of stay in the ED was significantly decreased for intervention groups (3 studies; standardized mean difference, −3.00 [95% CI, −3.61 to −2.39]; *I*^2^ = 99% [95% CI, 98%-99%]). There were no significant differences for other outcomes. Factors associated with outcomes included communication and referral processes between health care professionals.

**Conclusions and Relevance:**

Emerging evidence suggests that transitional care interventions are associated with lower readmissions for residents of LTCFs 65 years and older. Despite this and with aging populations, investment in such interventions has been remarkably low across most countries.

## Key Points

**Question:**

Are transitional care interventions associated with improved outcomes among residents of long-term care facilities (LTCF) who are 65 years and older?

**Findings:**

This systematic review and meta-analysis of data from 15 quantitative studies (32 722 participants or records) and 4 qualitative studies found that residents of LTCFs who are 65 years or older and were allocated to transitional care interventions were 1.7 times less likely to be readmitted to the hospital or the emergency department and had small reductions in length of stay in the emergency department compared with residents allocated to control groups.

**Meaning:**

These findings suggest that transitional care interventions are associated with reduced readmissions for residents of LTCFs who are 65 years or older, but such interventions are currently sparse and warrant investment from health service practitioners.

## Teaser

This systematic review and meta-analysis examines the association of transitional care interventions with hospital and emergency department readmissions for patients 65 years and older who are transitioning in and out of long-term care facilities and explores factors that potentially mitigate the outcomes and implementation of transitional care interventions.

## Introduction

Globally, the number of people 65 years and older is expected to double between 2019 and 2050 to 1.5 billion,^1^ resulting in a dramatic increase in the number of residents of long-term care facilities (LTCF). Residents of LTCFs (including nursing homes and residential aged care facilities) typically have multiple long-term conditions, have cognitive impairment, are twice as likely to experience unplanned hospital admissions compared with non-LTCF residents, and are more likely to be readmitted to the hospital.^2,3,4,5^ Of people 75 years and older in their last year of life, 81% have at least 1 hospitalization, and 96% have at least 1 emergency admission.^6^ Among residents of LTCFs, 67% of hospitalizations are potentially avoidable,^7^ and emergency department (ED) visits are associated with complications such as pressure ulcers, delirium, and infections.^8^ Reducing avoidable hospital readmissions has been the policy focus for residents of LTCFs before the COVID-19 pandemic, because US Medicare readmissions alone cost $24 billion annually, and unplanned readmissions cost $17.4 billion.^9,10^ During the pandemic, substantial decreases in the number of admissions and readmissions have been observed for people in LTCFs compared with previous years and compared with the general population.^11^ However, a universal reduction of readmissions (both avoidable and unavoidable) for people in LTCFs may unintentionally contribute to the exclusion of older people from health care and increase the risk of harm if patients are not admitted when clinically indicated.

Improving the quality of care for older people in LTCFs who transition from one care setting or level to another (transitional care) is a major challenge for health care systems in most developed countries. Quality transitional care has several dimensions, including communication between health care professionals around discharge assessment and care planning, preparation of the patient and caregiver for care transition, timely and complete exchange of information between all parties (staff in different settings, patients, family caregivers), staff training, and patient and caregiver education on self-management.^12,13,14,15^ Previous systematic reviews^16,17,18,19^ have focused on transitional care for community-based older adults, particular subtypes of transitional care interventions (eg, communication of medical information), or particular types of transitions (eg, from an LTCF to the ED). None, however, have focused on transitional care more broadly for older people transitioning in and out of LTCFs, despite residents of LTCFs having greater functional impairment than community-dwelling older adults,^20^ which adds to the complexity of transitional care. Furthermore, little is known about which factors are associated with the outcomes and implementation of transitional care interventions for residents of LTCFs. For example, greater cross-sector communication, focusing on all key informants of the transition process (residents, staff members), as well as the system (ie, care pathways), and the involvement of primary care and community practitioners may improve care and prevent admissions and readmissions. However, evidence is limited regarding the contribution of these factors in the success of transitional care interventions.^21,22,23,24,25^

We conducted a systematic review to (1) examine the association of transitional care interventions with outcomes for older people (≥65 years of age) transitioning into and out of LTCFs and staff and (2) explore factors that potentially mitigate the outcomes and implementation of transitional care interventions for residents of LTCFs. These factors include quality of engagement with community and primary care practitioners, multifocused design of the transitional care strategy, and communication across services.

## Methods

**Figure 1.**
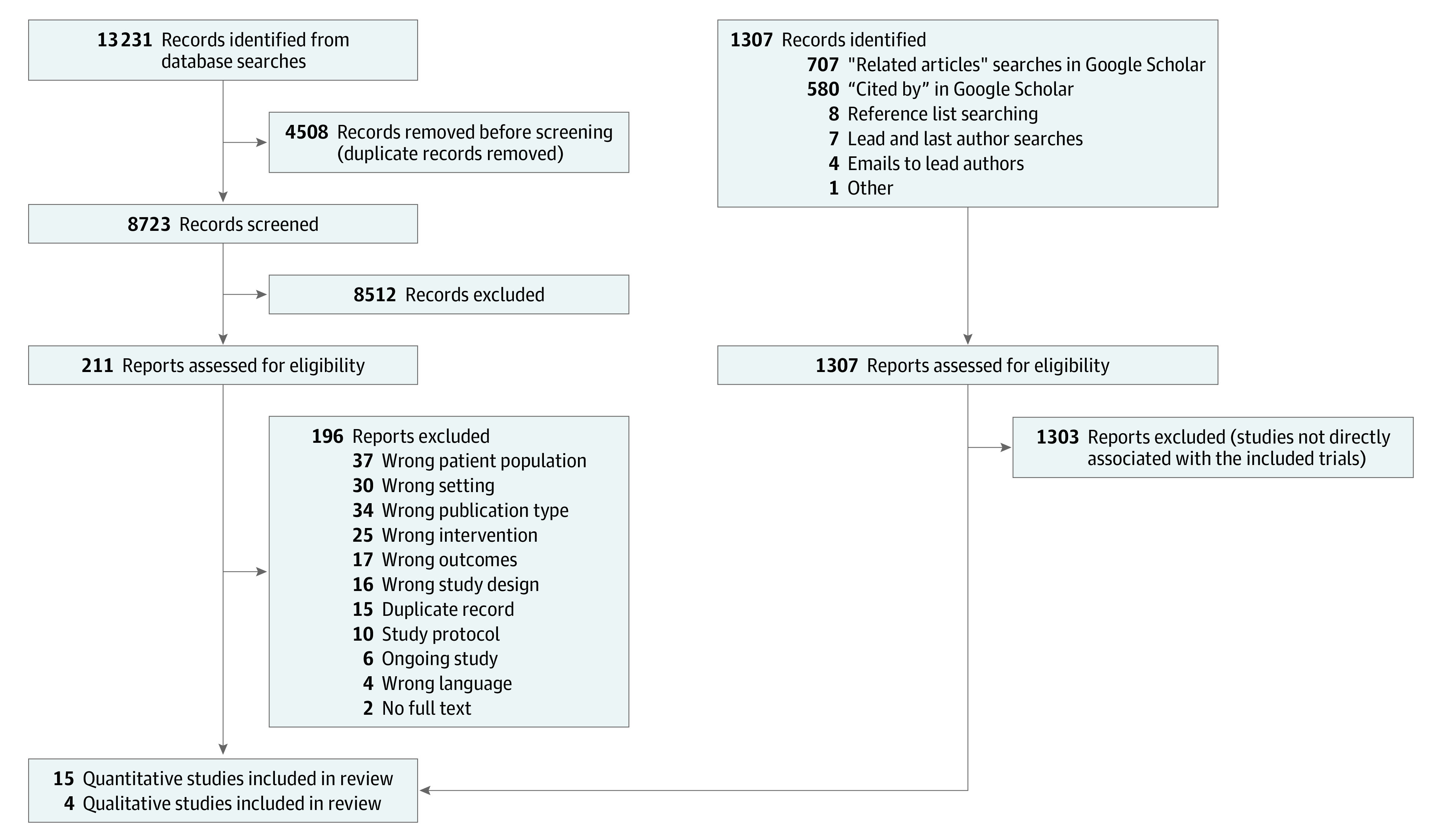
PRISMA Diagram of Study Selection An updated PRISMA guideline is found in Page et al.^26^

This systematic review and meta-analysis was conducted and reported in accordance with the Preferred Reporting Items for Systematic Reviews and Meta-analyses (PRISMA) reporting guideline. The PRISMA flow diagram is found in Figure 1.^26^ and is registered with the protocol on PROSPERO (CRD42021224313).

### Searches

With support from an information specialist, searches were undertaken in the following databases from inception until July 21, 2021: MEDLINE, EMBASE, PsycINFO, Cochrane Central Register of Controlled Trials, and Cumulative Index to Nursing and Allied Health Literature. Process evaluations and qualitative studies associated with the included trials were identified using aspects of the CLUSTER methodology (citations, lead authors, unpublished materials, searched Google Scholar, tracked theories, ancestry search for early examples, and follow-up of related projects)^27^ (further details are provided in the eMethods and eTable 1 in the Supplement).

### Eligibility Criteria

We included studies with controlled intervention designs as described in the Cochrane Handbook for Systematic Reviews of Interventions, version 6.2,^28^ which evaluated transitional care interventions for people 65 years and older living in LTCFs. Patient outcomes included 30-, 60-, and 90-day readmission rates to the hospital or ED (primary outcome), functional independence (Barthel score), health-related quality of life, knowledge of the care plan, medication adherence, adherence to follow-up, patient or caregiver satisfaction, person-centered care, symptom management, discharge readiness, and length of stay in days until discharge. Staff outcomes included job satisfaction, quality of interprofessional communication and teamwork, and well-being (eg, burnout). We excluded studies not targeting health care transitions and those not published in English. Studies involving a first-time transition to an LTCF were included if they evaluated the relevant outcomes.

### Data Selection and Extraction

Records were independently screened by 2 reviewers in 2 stages: (1) title and abstracts and (2) full texts. Disagreements were resolved through discussion and involvement of a third experienced reviewer. Interrater reliability scores were greater than 75%, reflecting moderate to strong levels of agreement.^29^ Three public contributors were involved in various stages of the review, including discussions of studies to be included (further details are provided in the eMethods in the Supplement).

We adapted the Cochrane Collaboration data extraction form^30^ and extracted the following information from included studies: population characteristics, intervention content and delivery method, study design, and outcomes. All data were extracted by one reviewer and checked by a second reviewer. One study author was contacted for clarification of sample sizes.^31^ Data extraction from associated qualitative studies was informed by “coding” in primary qualitative research.^32,33^ Two authors (A.H. and G.D.W.) read the articles in detail and recorded any possible explanations for intervention and outcome associations.

### Risk of Bias Assessment

Two independent reviewers applied the risk of bias assessment tool for randomized clinical trials (RCTs), the revised risk of bias tool for cluster RCTs,^34^ the ROBINS-I (Risk of Bias in Nonrandomized Studies of Interventions) tool for nonrandomized studies,^35^ and the Critical Appraisal Skills Programme Qualitative Studies checklist^36^ for the associated qualitative studies. Disagreements were resolved by discussion and consensus among the research team (see eTable 3 in the Supplement for the risk of bias results and definition of high risk of bias).

### Statistical Analysis

#### Quantitative Meta-analysis

The outcome data were converted to either the log odds scale or the standardized mean difference (SMD) using comprehensive meta-analysis software. Effect sizes were then pooled using DerSimonian-Laird random effects.^37^ Results were presented in the forest plots either through odds ratios (ORs) by exponentiating, or SMDs calculated using Hedges *g* and then interpreted according to Cohen’s criteria.^38^ Standardized mean differences were used owing to the various different scales of measurement that were applied for each of the continuous variables (ie, length of stay, quality of life, and Barthel score).^39^ Hartung-Knapp confidence intervals were used to account for uncertainty in the variance estimate.^40^ For mortality or adverse events, effects were assessed by pooling the relative risk (RR). These RRs were then pooled across trials using Mantel-Haenszel fixed-effect or inverse-variance random-effects meta-analysis depending on the number of studies reporting the outcome of interest. In meta-analyses involving fewer than 5 studies, the confidence intervals for the effect size based on the random-effects model are often too wide. The fixed-effects model can overcome this by favoring the studies with the largest weight and avoid estimating the between-study variance (as for the random-effects model), which can be low in accuracy when there are few studies.^41,42^ We performed 3 subgroup analyses to examine the association of involving primary care and community practitioners, having 1 or more areas of focus for the intervention (eg, resident, system, or staff), and country of origin (this was a post hoc analysis of studies based in Australia vs elsewhere), and a sensitivity analysis retaining only studies at low risk of bias in the readmissions outcome.

Heterogeneity was quantified using the *I*^2^ statistic with values of 25% indicating low, 50% indicating moderate, and 75% indicating high heterogeneity.^43^ For each meta-analysis with 10 studies or more, funnel plots and Begg and Egger tests were used to examine potential for publication bias.^44^ The trim-and-fill method was used as a sensitivity analysis to observe causes of small study publication bias. Cluster RCTs were analyzed by adjustment using a sample size and variation inflation method, assuming an intraclass correlation of 0.02.^45^ When studies reported more than 1 control group, these were combined using the formulas described in the Cochrane Handbook for Systematic Reviews of Interventions, version 6.2.^28^ All meta-analyses were conducted in R, version 4.0.3 (R Foundation for Statistical Computing) using the meta or metafor packages.^46,47^ Two-sided *P* < .05 indicated statistical significance.

#### Qualitative and Integrative Synthesis

An overarching theme was developed by the last author (G.D.-W.) from the extracted findings,^32^ incorporating key factors that were commensurate across the published studies. Findings were written as a narrative description highlighting the main issues. The theme was discussed and refined with the fourth author (A.H.), and after completion of the meta-analysis, the whole study team met to discuss the extent to which the qualitative findings explained any variation found in the quantitative results.

## Results

Of 14 538 records identified from the searches, 15 quantitative studies^31,48,49,50,51,52,53,54,55,56,57,58,59,60,61^ (totaling 32 722 participants and records), and 4 associated process evaluations and qualitative studies^62,63,64,65^ were included (the PRISMA flow diagram is presented in Figure 1). Excluded studies are listed in eTable 2 in the Supplement.

### Characteristics of Studies, Populations, Interventions, and Outcomes

Table 1 presents the 15 included intervention studies,^31,48,49,50,51,52,53,54,55,56,57,58,59,60,61^ published between 2002 and 2020. Ten studies were set in Australia,^31,48,49,50,51,52,53,54,55,56^ 3 in the United States,^57,58,59^ 1 in Hong Kong,^60^ and 1 in Denmark.^61^ Six studies were RCTs,^48,50,51,53,57,59^ including 1 cluster RCT^57^ and 1 prospective RCT^48^ (the full details of the study designs are provided in Table 1). The comparator for 14 of the studies^31,48,49,50,51,52,53,54,55,56,58,59,60,61^was usual care, policy, and/or process, and 1 study^57^ included both a usual care control group and an attention control group that involved sharing information about attempts to reduce hospitalizations. Of the 4 linked qualitative studies, 2 were exploratory qualitative studies completed in advance of the main trial to inform intervention design,^62,63^ and 2 were process evaluations that occurred contemporaneously with the trials.^64,65^

**Table 1.** Characteristics of Included Studies

Source (country)	Design	Sample size	Brief intervention description	Who delivered the intervention	Focus of intervention	Involvement of primary care or community clinician	Time point of intervention	Direction of transfer
Cordato et al,^48^ 2018 (Australia)	Prospective RCT	45	Regular Early Assessment Post-Discharge intervention. Conjoint geriatrician and nurse practitioner evaluations (involving cognition, medication use, and quality of life) for 6 mo after discharge.	Geriatrician and nurse practitioner	Resident focused	REAP clinicians advise GPs on investigations and treatments.	After discharge	Hospital to LTCF
Crilly et al,^49^ 2011 (Australia)	Nonrandomized clincial trial	177	HINH program involving acute nursing support, provision of equipment, training and education for staff, regular checks on patient progress by an HINH nurse.	Aged care facility nursing staff; HINH nurse	Mix: staff and system focused	None	At admission	Hospital to LTCF
Crotty et al,^50^ 2004 (Australia)	RCT	110	Pharmacist transition coordinator coordinated medication transfer summaries from hospital, medication reviews, case conferences with physicians and pharmacists.	Pharmacist	System focused	Family physicians and community pharmacists sent extra information.	Multiple: before and after discharge	Hospital to LTCF
Crotty et al,^51^ 2005 (Australia)	RCT	320	Off-site care facility for patients awaiting assessment and transfer to a care home.	Hospital and private care clinician	System focused.	None	Multiple: after discharge and during transition	Hospital to LTCF
Elliott et al,^52^ 2012 (Australia)	Prospective preintervention-postintervention study	593	Pharmacist-prepared IRCMAC sent with the patient from the hospital to the care facility.	Hospital pharmacist	System focused	None	Multiple: after discharge and during transition	Hospital to LTCF
Harvey et al,^53^ 2014 (Australia)	RCT	123	Outreach service: assessment and development of care plan, advance care plan discussions with patients and families, intercurrent illness management reviews, education and support for care facility staff and primary care physician.	Geriatrician and aged care nurse consultant	Mix: resident and staff focused	Primary care physician received education and support	After discharge	Hospital to LTCF
Hullick et al,^31^ 2016 (Australia)	Controlled preintervention-postintervention design	413	Aged Care Emergency Service: clinical care manual, nurse-led telephone triage line, education, case management, development of collaborative relationships.	ED advanced practice nurse; ED registered nurse	System focused	None	Before admission	LTCF to hospital
Kane et al,^57^ 2017 (US)	Cluster RCT (implementation trial)	23 478	Tools to identify changes in patients, document staff communication, care paths, project champions. Training, telephone support and webinars for staff (to support implementation of INTERACT).	Study team and nursing home staff	Mix: staff and system focused	None	Before admission	LTCF to hospital
Layton,^58^ 2019 (US)	Quasi-experimental, 2-group design	38	CHF-specific education and protocols for nursing home staff: education on documentation, care plan implementation, assessment and skills.	Educational intervention delivered to frontline nursing home staff (eg, registered nurses, nursing assistants)	Staff focused	None	Intervention for staff was before admission, relevant to patients after discharge	LTCF to hospital
Lee et al,^60^ 2002 (Hong Kong)	Matched, randomized case-control trial	89	Postdischarge care protocol, education for nursing home staff, information sharing with patients and staff, individualized care planning, telephone support.	Delivered by community nursing staff to nursing home staff and patients	Mix: staff and resident focused	Community nurses provided support to nursing home staff.	Begins after initial discharge and can be before and after subsequent readmissions	Hospital to LTCF
Mudge et al,^56^ 2012 (Australia)	Controlled trial	1004	Model of care involving greater and consistent staffing, structured daily interdisciplinary meetings, explicit discharge planning.	Clinical staff based at the hospital	System-focused model of care	None	Before discharge	Hospital to LTCF
Mukamel et al,^59^ 2016 (US)	RCT	225	Reengineered discharge process and app to support patient selection of nursing home. App included an educational module and a preference elicitation module.	Project coordinator provided iPad; intervention delivered via app	Resident focused	None	Before discharge	Hospital to LTCF
Pedersen et al,^61^ 2018 (Denmark)	Quasi-randomized study	648	Individualized postdischarge support: assessment of clinical condition, medication, discussions with the patient, relatives, and nursing home staff. In-person and telephone support.	Physician and nurse from a geriatric team	Resident focused	None	After discharge only	Hospital to LTCF
Shrapnel et al,^54^ 2019 (Australia)	Preintervention-postintervention study	1130	HINH-inspired model of care. Clinical liaison with care facility staff and GP clinicians, acute care management, shared accountability for care.	Specialist nurse	Mix	Hospital-based nurses worked in partnership with GP clinicians.	Before admission, at admission, before discharge	Both LTCF to hospital and hospital to LTCF
Street et al,^55^ 2015 (Australia)	Preintervention-postintervention study	4329	Residential In-Reach service: skilled assessment and diagnostic support to care facility staff, telephone advice and triage, in-person support, education, and training for staff.	Specialist practice nurses, supported by a geriatrician	Mix	None	Before admission	LTCF to hospital

Abbreviations: app, application; CHF, congestive heart failure; ED, emergency department; GP, general practitioner; HINH, Hospital in the Nursing Home; INTERACT, Interventions to Reduce Acute Care Transfers; IRCMAC, interim residential care medication administration chart; LTCF, long-term care facility; RCT, randomized controlled trial.

The quantitative studies included data from 32 722 participants and/or records. The median pooled sample across the studies was 320 (range, 38-23 478). The mean age of participants ranged from 75^59^ to 90 years.^48^ With the exception of 4 studies,^54,58,60,61^ participants were not recruited according to health condition.

Transitional care interventions had several components such as discharge planning and/or postdischarge communication and support, new models and/or pathways of care, training and education for staff members in LTCFs or hospitals, and medication reviews (Table 1). Ten interventions^48,49,50,51,52,53,56,59,60,61^ were evaluated while patients were transitioning from hospitals to LTCFs, whereas 4 interventions^31,55,57,58^ were evaluated while patients were transitioning from LTCFs to hospitals, and 1 intervention^54^ was evaluated during both types of transition. The interventions were delivered at multiple time points (Table 1) and focused on the system (5 studies),^31,50,51,52,56^ residents (3 studies),^48,59,61^ staff (1 study),^58^ or a combination (6 studies).^49,53,54,55,57,60^ Where reported (in 12 studies),^31,48,50,51,52,54,55,57,58,59,60,61^ intervention duration ranged from a few hours for a resident-focused intervention^59^ to 2 years for an intervention that included a change to a service.^55^ Interventions were delivered by nurses based at hospitals or LTCFs or pharmacists, physicians and other health care practitioners who often were transition coordinators. Four studies^48,50,53,54^ involved community or primary care practitioners who received additional information, advice, and support as part of the intervention. In 1 study,^60^ community nurses delivered the intervention, following a protocol to care for patients and educating nursing home staff about the care of the patient. Family members and caregivers tended not to play an active role in the interventions. Where they were involved, typically they were recipients of information or supported the resident during decision-making.^53,59,61^ In 1 study,^54^ the intervention focused on residents, staff, and the system (ie, a model of care), and families could access social support as part of the intervention.

As per inclusion criteria, 14 studies reported data on readmissions (11 studies on hospital readmissions^31,48,49,50,51,52,53,56,57,60,61^ and 5 on ED readmissions^31,48,54,55,58^), 6 studies^48,51,53,55,56,61^ reported data on all-cause mortality, 7 studies^31,48,49,53,56,59,60^ reported hospital length of stay, and 3 studies^31,49,60^ reported ED length of stay. Only 2 studies^51,60^ reported data on patient quality of life and functional status. Only 1 study^50^ reported adverse events, for which the difference between groups was reported as nonsignificant (*P* = .58). No other patient or staff outcomes were reported across the studies.

### Risk of Bias Results

The quality of the studies was variable (eTable 3 in the Supplement). Six studies (40%)^48,50,51,53,57,61^ had a low risk of bias for the random sequence generation, and 3 studies (20%)^50,51,53^ had low risk for allocation concealment. Blinding of patients, outcome assessors, and analysts was poorly reported across most studies. The 4 qualitative studies^62,63,64,65^ met 6 to 9 items from the Critical Appraisal Skills Programme Qualitative Studies checklist,^36^ indicating they were medium to high quality^66^ (eTable 4 in the Supplement).

### Meta-analysis

**Figure 2.**
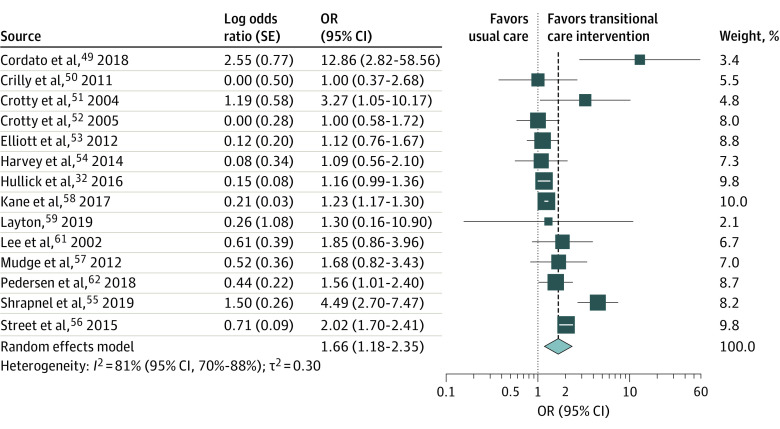
Forest Plot of Hospital and Emergency Department Readmissions Combined OR indicates odds ratio; error bars, 95% CI; and diamond marker, heterogeneity.

**Figure 3.**
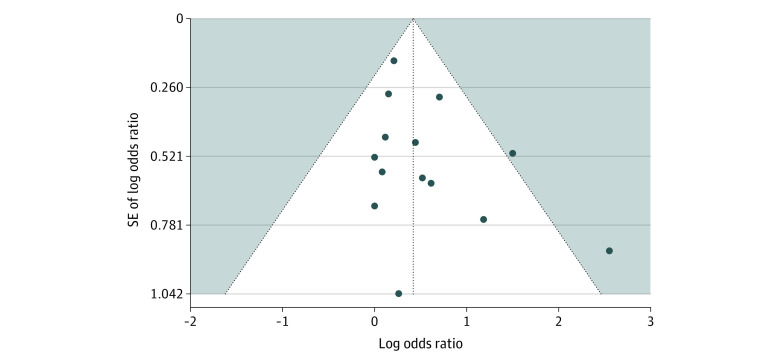
Readmissions Funnel Plot of Statistical Tests of Publication Bias For the classic Egger test for funnel plot asymmetry, *t*_12_ = 1.656 (*P* = .12). For the mixed-effects version of the Egger test, *z* = 1.763 (*P* = .08). For the trim-and-fill test, *z* = 3.387 (*P* < .001).

Older people allocated to transitional care interventions were 1.7 times less likely to be readmitted in hospitals or ED compared with those allocated to control groups (14 studies^31,48,49,50,51,52,53,54,55,56,57,58,60^; OR, 1.66 [95% CI, 1.18-2.35]; *I*^2^ = 81% [95% CI, 70%-88%]) (Figure 2). There was no evidence of funnel plot asymmetry (Figure 3), and results of the Eggers test were nonsignificant (*t*_12_ = 1.656; *P* = .12).

A significant difference was found between the intervention and the control groups for reduction in readmissions to hospitals (11 studies^31,48,49,50,51,52,53,56,57,60,61^; OR, 1.48 [95% CI, 1.01-2.17]; *I*^2^ = 40% [95% CI, 0%-66%]) (eFigure 6 in the Supplement) and for length of stay in ED (3 studies^31,49,60^; SMD, −3.00 [95% CI, −3.61 to −2.39]; *I*^2^ = 99% [95% CI, 98%-99%]) (eFigure 1 in the Supplement). No significant differences were found for readmissions to the ED (5 studies^31,48,54,55,58^; OR, 2.04 [95% CI, 0.96-4.33]; *I*^2^ = 93% [95% CI, 87%-96%]) (eFigure 6 in the Supplement), all-cause mortality (6 studies^48,50,53,55,56,61^; RR, 0.95 [95% CI, 0.79-1.16]; *I*^2^ = 0 [95% CI, 0-75%]) (eFigure 4 in the Supplement), length of stay in hospital (7 studies^31,48,49,53,56,59,60^; SMD, −1.86 [95% CI, −5.47 to 1.75]; *I*^2^ = 98% [95% CI, 97%-99%]) (eFigure 1 in the Supplement), quality of life (2 studies^50,60^; SMD, −0.04 [95% CI, −0.46 to 0.38]; *I*^2^ = 92% [95% CI, 72%-98%]) (eFigure 2 in the Supplement), and functional independence (Barthel score) (2 studies^50,60^; SMD, −0.83 [95% CI, −1.25 to −0.41]; *I*^2^ = 85% [95% CI, 39%-96%]) (eFigure 3 in the Supplement) (also see Table 2).

**Table 2.** Results of All Meta-analyses

Outcome (model)	No. of studies	No. of patients	Effect size (95% CI)	*I*^2^ (95% CI), %
Combined hospital and ED readmissions (random-effects)	14	32 497	OR, 1.66 (1.18 to 2.35)	81 (70 to 88)
Length of stay in hospital (random-effects)	7	2076	SMD, −1.86 (−5.47 to 1.75)	98 (97 to 99)
Length of stay in ED (fixed-effect)	3	679	SMD, −3.00 (−3.61 to −2.39)	99 (98 to 99)
Quality of life (fixed-effect)	2	409	SMD, −0.04 (−0.46 to 0.38)	92 (72 to 98)
Barthel score (fixed-effect)	2	409	SMD, −0.83 (−1.25 to −0.41)	85 (39 to 96)
All-cause mortality (random-effects)	6	6469	RR, 0.95 (0.79 to 1.16)	0 (0 to 75)

Abbreviations: ED, emergency department; OR, odds ratio; RR, relative risk; SMD, standardized mean difference.

In line with the main analysis, the sensitivity analysis removing the high risk of bias studies showed fewer readmissions in those receiving the intervention (3 studies^51,54,57^; OR, 1.25 [95% CI, 1.19-1.31]; *I*^2^ = 92% [95% CI, 80%-97%]) (eFigure 10 in the Supplement). The subgroup analyses showed that neither the involvement of primary care or community practitioners and the multifocus design significantly influenced the association between transitional care interventions and reduced readmissions (eFigures 7 and 8 in the Supplement). The post hoc subgroup analysis showed that interventions in Australian studies were associated with fewer readmissions (10 studies^31,48,49,50,51,52,53,54,55,56^; OR, 1.78 [95% CI, 1.06-3.00]) compared with interventions based elsewhere (4 studies^57,58,60,61^; OR, 1.24 [95% CI, 1.18-1.30), but this difference was not significant (eFigure 9 in the Supplement).

### Qualitative Findings

#### Miscommunication and Mismatched Expectations

The analysis of the associated qualitative studies identified 3 key factors that may influence the association between transitional care interventions and outcomes, including the quality of communication and role clarity of staff members across involved settings, quality of information flow and referral pathways, and engaging community and primary care practitioners. These factors appeared to underpin a broader theme regarding (at times mismatched) expectations and understandings between stakeholders of each other’s skills and knowledge.

There appeared to be stereotypes or expectations of different staff groups (eg, in relation to their clinical skills). Thus, 1 study^62^ reported that care assistants felt that hospital staff had unrealistic expectations of their skills. On the other hand, LTCF staff in another study^63^ felt that ED staff did not know how to properly care for acutely unwell people with dementia. However, the same study^63^ also reported that LTCF staff felt they were “out of their depth” when faced with acutely unwell residents. Further, ED staff appeared dismissive of clinical information provided by LTCF staff, because of their employment grade and training.^63^ This finding points to further opportunities for interventions to work on different areas when attempting to smooth care transitions (see Discussion). The clinical (and other) skills of the personnel involved in the intervention were also important, with 1 study^49^ reporting that the intervention lead needed to be both clinically skilled as well as aware of local health and community services.

Three of the 4 studies^62,63,64^ pointed to the importance of involving primary care and community clinicians in aiding intervention processes. Care facility staff appeared more confident about making decisions when there was a primary care physician (or ambulance crew) to defer to.^63^

## Discussion

### Summary of Main Findings

This systematic review of 15 controlled interventions found that residents of LTCFs 65 years and older allocated to transitional care interventions were 1.7 times less likely to be readmitted to hospitals or EDs, experienced significantly fewer hospital readmissions, and had shorter ED length of stay compared with residents allocated to usual care. Transitional care interventions were not associated with improvements in any other patient or staff outcomes, but this finding predominately reflects the low quality of outcome capture and/or reporting across the included studies rather than absence of improvements. The qualitative findings identified a broad theme of miscommunication and mismatched expectations between stakeholders.

### Comparison With Previous Studies

Our findings are consistent with those of a recent systematic review^67^ that evaluated the impact of transitional care interventions on readmissions for older people transitioning from hospital to home living in the community. This review found some evidence that high-intensity transitional care interventions (with a greater number of components and longer duration) are associated with reduced hospital readmissions, but most studies were of poor quality. Moreover, 1 review of interdisciplinary interventions in nursing homes^68^ reported that all trials involving a primary care physician or a pharmacist were successful, and another systematic review exploring the appropriateness of transferring nursing home residents to EDs^24^ recommended greater involvement of primary care practitioners. Other systematic reviews and empirical studies^69,70,71,72^ have highlighted the need for better communication and coordination across care settings (eg, provision of more complete information and tools to support this), which echoed our qualitative findings. Our public contributors also queried the roles and training of staff and felt clarity regarding what certain staff groups (eg, pharmacists) can and cannot do was lacking. Consistent with this, family member and medical practitioner perceptions (or misperceptions) about the level of care that could be provided by nursing home staff acted as a barrier to implementation of the INTERACT (Interventions to Reduce Acute Care Transfers) program to reduce hospitalizations.^65^

### Implications for Clinicians, Policy Makers, and Researchers

With the exception of Australia, clinicians and policy makers across most developed countries (including the US, UK, and Europe) with a fast-growing proportion of aged populations should note the striking lack of investment in transitional care interventions for older people living in LTCFs. Transitional care interventions function at the juncture of 2 or more separate services that may be operating in extremely complex health and social care systems in which there is ambiguity over responsibility and accountability (eg, in England^73^). This means that the implementation of such interventions may be particularly complex and warrants further attention. Older people living in LTCFs experience fewer readmissions after their participation in transitional care interventions compared with usual care. It is therefore essential to invest in transitional care interventions owing to the high human and financial cost of avoidable readmissions in this vulnerable group of people.^72^ There might be opportunities to improve transitional care interventions for older people living in LTCFs by (1) promoting high-quality communication and role clarity among staff members across involved settings and (2) building the infrastructure for better information flow and referral pathways. Findings may also be transferable to other populations who experience transitions of care, for example people younger than 65 years with complex care needs, community residents, or adolescents transitioning from child to adult services.

Future trials of transitional care interventions would benefit from a comprehensive core set of outcomes to better capture impacts on patient experience and safety, staff outcomes, and systemic costs as well as consistent follow-up time points to improve data comparability and pooling. In addition, embedding adherence guides to improve staff and resident and caregiver engagement while evaluating or implementing transitional care interventions is recommended.

### Limitations

To our knowledge, this is the first systematic review of transitional care interventions among older people living in LTCFs in which both meta-analysis and integration of associated qualitative studies have been applied. However, this study has some important limitations. The overall quality of the studies was low, and there was considerable variation in the content of interventions, the time point of intervention delivery, and the follow-up assessment time points. Although we used appropriate methods to account for and explore the heterogeneity (ie, random-effects models and subgroup and sensitivity analyses), it is likely that important sources of heterogeneity remain undetected. In addition, not all analyses originally proposed in the review protocol were performed owing to poor data capture and reporting, including analyses on subjective patient experience, adverse events, and staff outcomes. The lack of data on adverse events is of particular concern, because almost 4 in 10 transfers from hospitals to LTCFs have associated adverse events, more than 70% of which could be prevented or ameliorated.^74^ Because two-thirds of the studies were based in Australia, the transferability of the findings to other developed countries may be limited. Finally, although our qualitative findings showed that involvement of primary care and community practitioners may be associated with the outcomes and implementation of the transitional care interventions, the subgroup analysis did not show a significant difference between interventions involving primary care and community practitioners and those that did not. These nonsignificant findings may reflect the small number of studies included in this subgroup analysis and warrant further exploration once more data on transitional care interventions are available. Furthermore, these findings may also reflect an inappropriate choice of primary outcome measure. Readmission was selected as the primary outcome for this review because it is the most commonly reported outcome in studies of transitional care interventions, and other quantitative outcomes are rarely reported. However, the use of readmissions to measure outcomes has been subject to much debate, and other health status or quality of life measures may be more appropriate.^75,76^

## Conclusions

This systematic review and meta-analysis found that transitional care interventions are associated with reduced readmissions to hospital or ED for residents of LTCFs who are 65 years or older. Most developed countries with aging populations, including the US, Europe, and UK, have no national policy or investment in transitional care interventions for people living in LTCFs. While developing and implementing such interventions, careful consideration is recommended on the quality of communication and role expectations of staff members across settings, and the availability of infrastructure and technology to enable information flow. Finally, it is important for transitional care interventions to demonstrate their effectiveness in improving an agreed set of important outcomes for patients, staff members, and the health and care systems.

## Notes

Supplement. **eMethods.** Search Strategy, Eligibility Criteria, and Patient and Public Involvement **eTable 1.** MEDLINE Search Terms **eTable 2.** Excluded Studies **eTable 3.** Risk of Bias Results **eTable 4.** CASP Scores for Qualitative Studies and Process Evaluations **eFigure 1.** Hospital and ED Length of Stay **eFigure 2.** Quality of Life **eFigure 3.** Barthel Score **eFigure 4.** Mortality Risk Ratio **eFigure 5.** Mortality Risk Difference **eFigure 6.** Subgroup Analysis of Hospital vs ED Readmissions **eFigure 7.** Subgroup Analysis of Readmission by Involvement of Primary Care **eFigure 8.** Subgroup Analysis by Focus of Intervention **eFigure 9.** Subgroup Analysis by Country **eFigure 10.** Sensitivity Analysis: Readmission by Risk of Bias
eReferences
