# Supplementary material for: Transitional Care Interventions for Older Residents of Long-term Care Facilities: A Systematic Review and Meta-analysis
Source: JAMA Netw Open. 2022 May 4;5(5):e2210192. doi: 10.1001/jamanetworkopen.2022.10192 (PMC9069255; doi:10.1001/jamanetworkopen.2022.10192)
Supplement: Supplement. — eMethods. Search Strategy, Eligibility Criteria, and Patient and Public Involvement eTable 1. MEDLINE Search Terms eTable 2. Excluded Studies eTable 3. Risk of Bias Results eTable 4. CASP Scores for Qualitative Studies and Process Evaluations eFigure 1. Hospital and ED Length of Stay eFigure 2. Quality of Life eFigure 3. Barthel Score eFigure 4. Mortality Risk Ratio eFigure 5. Mortality Risk Difference eFigure 6. Subgroup Analysis of Hospital vs ED Readmissions eFigure 7. Subgroup Analysis of Readmission by Involvement of Primary Care eFigure 8. Subgroup Analysis by Focus of Intervention eFigure 9. Subgroup Analysis by Country eFigure 10. Sensitivity Analysis: Readmission by Risk of Bias eReferences [file jamanetwopen-e2210192-s001.pdf]

Supplementary Online Content

Birtwell K, Planner C, Hodkinson A, et al. Transitional care interventions for older residents of long-term care facilities: a systematic review and meta-analysis. *JAMA Netw Open*. 2022;5(5):e2210192. doi:10.1001/jamanetworkopen.2022.10192

eMethods. Search Strategy, Eligibility Criteria, and Patient and Public Involvement
eTable 1. MEDLINE Search Terms
eTable 2. Excluded Studies
eTable 3. Risk of Bias Results
eTable 4. CASP Scores for Qualitative Studies and Process Evaluations
eFigure 1. Hospital and ED Length of Stay
eFigure 2. Quality of Life
eFigure 3. Barthel Score
eFigure 4. Mortality Risk Ratio
eFigure 5. Mortality Risk Difference
eFigure 6. Subgroup Analysis of Hospital vs ED Readmissions
eFigure 7. Subgroup Analysis of Readmission by Involvement of Primary Care
eFigure 8. Subgroup Analysis by Focus of Intervention
eFigure 9. Subgroup Analysis by Country
eFigure 10. Sensitivity Analysis: Readmission by Risk of Bias
eReferences

This supplementary material has been provided by the authors to give readers additional information about their work.

eMethods 1. Search Strategy, Eligibility Criteria, and Patient and Public Involvement

The search strategy uses three concept blocks:

Block A = Setting
Block B= Intervention
Block C= Study design

The search carried out was A + B + C. The strategy was developed for Medline (OVID) and then translated to Embase, PsycINFO, Cochrane Central Register of Controlled Trials (CENTRAL), and CINAHL. The Medline search terms can be found in eTable 1.

Process evaluations and qualitative studies associated with the included intervention trials were identified by employing the following elements of the CLUSTER methodology[1]: i) emailing first authors; ii) searching reference lists of included studies; iii) using the "cited by" and "related articles" features of Google Scholar; and iv) searching PubMed for other articles published by first and last authors of included studies. The full CLUSTER methodology was not employed as we were only searching for articles specifically associated with the included trials and not the wider contextual literature.

Eligibility criteria for quantitative studies in this review:

Population. People aged 65 and over living in LTCFs for older people, including facilities employing nurses or medical staff (e.g. nursing homes), and those providing assistance with personal care (e.g. residential care homes). Participants were not excluded on the basis of psychiatric or physical comorbidity.
Intervention. We included the following: i) Transitional interventions from an inpatient hospital unit/emergency department to a LTCF (e.g., individualized discharge plan, individualized transition record, post-discharge telephone follow-up, home visits, patient- and family-tailored discharge information, transition need assessment, reconciliation of medications at transition, a plan for follow-up, and patient education about self-management.). ii) LTCF-based interventions to prevent or prepare for re-admission to hospital. iii) Interventions delivered at the point of transition.
Comparators. Treatment as usual, enhanced usual care, or an active treatment comparator.
Outcomes. Patient outcomes included: 30-, 60- and 90-day readmission rates to hospital or emergency departments, or a combination (primary outcome), functional status, health-related quality of life, knowledge of care plan, medication adherence, adherence to follow-up, patient/carer satisfaction, person centred care, symptom management, discharge readiness, length of stay/day until discharge. Staff outcomes included: job satisfaction, quality of inter-professional communication/teamwork, well-being (e.g. burnout).
Design. Controlled intervention designs including randomized controlled trials (cluster, parallel, stepped wedge, factorial and crossover RCTs), non-randomized trials, controlled before-after studies, retrospective studies with historical controls and interrupted time series. We included associated process evaluation and qualitative studies associated to the main trial reports.
Setting. Any healthcare setting worldwide.
Exclusion criteria. Studies not written in English; conference abstracts and grey literature; interventions not specifically targeting healthcare transitions.

Patient and Public Involvement

Three Public Contributors (two male, one female) were involved at various stages of the review. Two of the Public Contributors had family members residing in care homes and the other was a carer for a community-dwelling family member. One Public Contributor was involved in six discussions during the development of the review protocol and made suggestions for types of studies that might be of interest. Three Public Contributors attended a meeting to discuss the included studies, intervention characteristics, outcomes and implications. In addition, one Public Contributor will be invited to co-write a lay summary of the findings. In line with the UK Standards for Public Involvement[189] and the Cochrane Statement of Principles[190], flexible opportunities for involvement were created and contributors were remunerated for their time. Following good practice recommendations, summary information was provided to contributors in advance of meetings where required[191,192].

© 2022 Birtwell K et al. *JAMA Network Open*.

eTable 1. MEDLINE Search Terms

Search terms	Search block	Source
1 Nursing Homes/ or nursing home*.mp.<br>2 Homes for the Aged/ or home* for the aged.mp.<br>3 Residential Facilities/ or Assisted Living Facilities/ or Group Homes/ or Halfway houses/<br>4 care home*.mp.<br>5 (aged adj2 (care or nursing or healthcare or residential) adj2 (facility or facilities or home*)).ti,ab.<br>6 ((geriatric or elderly) adj2 (facility or facilities or care home*)).ti,ab.<br>7 Hospitals, Veterans/<br>8 1 or 2 or 3 or 4 or 5 or 6 or 7<br>9 ((care or convalescent) adj (home* or center* or centre* or facility or facilities)).ti,ab.<br>10 ((skilled or intermediate) adj (nursing facility or nursing facilities)).ti,ab.<br>11 (resident* adj2 (care or facility or facilities)).ti,ab.<br>12 ((nursing or group or residential) adj home*).ti,ab.<br>13 ((longterm or long term) adj3 (care or facility or facilities)).ti,ab.<br>14 (healthcare adj2 (facility or facilities)).ti,ab.<br>15 assisted living.ti,ab.<br>16 group home.ti,ab.<br>17 halfway house.ti,ab.<br>18 sheltered accommodation.ti,ab.<br>19 9 or 10 or 11 or 12 or 13 or 14 or 15 or 16 or 17 or 18<br>20 exp aged/<br>21 geriatrics/<br>22 Veterans/<br>23 aged/ or "aged, 80 and over"/ or frail elderly/<br>24 (gerontol* or ageing or aging or elder* or geriatric* or seniors or old age or older or late* life).ti,ab.<br>25 (older adj (person* or people or adult* or patient* or inpatient* or outpatient*)).ti,ab.<br>26 veteran*.ti,ab.<br>27 20 or 21 or 22 or 23 or 24 or 25 or 26<br>28 19 and 27<br>29 8 or 28	Care homes / older adults	Adapted from Alldred (2016)[2]
30 Continuity of Patient Care/<br>31 Transitional Care/<br>32 transition*.ti,ab.<br>33 (transfer* or transferred or transferral or transferring).ti,ab.<br>34 (transition* adj10 (care or service* or center* or centre* or clinic* or facility or facilities or unit* or department* or patient*)).ti,ab.<br>35 ((transfer* or transferred or transferral or transferring) adj10 (care or service* or center* or centre* or clinic* or facility or facilities or unit* or department* or patient*)).ti,ab.<br>36 30 or 31 or 32 or 33 or 34 or 35	Care transitions	Adapted from Campbell (2016)[3] and Murray (2019)[4]

© 2022 Birtwell K et al. *JAMA Network Open*.

37 Patient Discharge/<br>38 Patient Transfer/<br>39 Patient Care Planning/ or case management/<br>40 "Delivery of Health Care, Integrated"/<br>41 Decision support systems, clinical/<br>42 Patient education/<br>43 Geriatric assessment/<br>44 Medication Reconciliation/<br>45 37 or 38 or 39 or 40 or 41 or 42 or 43 or 44<br>46 (continuity adj3 (care or health care or healthcare or treatment* or therapy or therapies or patient* or doctor* or nurse*)).ti,ab.<br>47 shared care.ti,ab.<br>48 shared service*.ti,ab.<br>49 ((healthcare or care or service*) adj3 integrat*).ti,ab.<br>50 (discharge and (plan* or service* or program* or intervention*)).ti,ab.<br>51 (patient* adj2 discharge*).ti,ab.<br>52 (hospital adj2 discharge*).ti,ab.<br>53 (discharge adj2 plan*).ti,ab.<br>54 (discharge adj service*).ti,ab.<br>55 (discharge adj program*).ti,ab.<br>56 (discharge adj procedure*).ti,ab.<br>57 46 or 47 or 48 or 49 or 50 or 51 or 52 or 53 or 54 or 55 or 56<br>58 "Length of Stay"/<br>59 Patient Admission/ (<br>60 Patient Readmission/<br>61 (readmission or readmitted or re-admission or re-admitted).ti,ab.<br>62 (rehospitali*ation* or re-hospitali*ation* or rehospitali*ed or re-hospitali*ed).ti,ab.<br>63 length of stay.ti,ab.<br>64 length of hospital stay.ti,ab.<br>65 patient admission.ti,ab.<br>66 ((hospital or hospitali*ed or bed) adj2 days).ti,ab.<br>67 58 or 59 or 60 or 61 or 62 or 63 or 64 or 65 or 66<br>68 36 or 45 or 57 or 67		
69 randomized controlled trial.pt.<br>70 controlled clinical trial.pt.<br>71 randomized.ab.<br>72 Clinical Trial/<br>73 randomly.ab.<br>74 trial.ti.<br>75 intervention.ti.<br>76 69 or 70 or 71 or 72 or 73 or 74 or 75<br>77 (pre post or "pre test*" or pretest* or posttest* or "post test*" or (pre adj5 post)).ti,ab.<br>78 ("quasi experiment*" or quasiexperiment* or "quasi random*" or quasirandom* or "quasi control*" or quasicontrol* or ((quasi* or experimental) adj3 (method* or study or trial or design*))).ti,ab.<br>79 ("time series" adj2 interrupt*).ti,ab.<br>80 (multicentre or multicenter or multi centre or multi center).ti,ab.<br>81 (random* or controlled).ti,ab.	Study design	Cochrane sensitivity and precision maximising version (2011)[5]

© 2022 Birtwell K et al. *JAMA Network Open*.

82	77 or 78 or 79 or 80 or 81		
83	76 or 82		
84	29 and 68 and 83		

© 2022 Birtwell K et al. *JAMA Network Open*.

eTable 2. Excluded Studies

Study	Reason for exclusion
Ackermann et al.,[6] 1998	Wrong outcomes
Adrion et al.,[7] 2020	Wrong patient population
Aizen et al.,[8] 2001	Wrong intervention
Aizen et al.,[9] 2014	Wrong language
Akinjogbin et al.,[10] 2020	Wrong publication type
Allen et al.,[11] 2011	Study protocol
Almehlisi et al.,[12] 2019	Wrong patient population
Altfeld et al.,[13] 2012	Wrong setting
Arendts et al.,[14] 2018	Wrong outcomes
Arendts,[15] 2019	Wrong outcomes
Arendts,[15] 2019	Duplicate record
Armagan et al.[16], 2004	Wrong patient population
Bader,[17] 2014	Wrong setting
Barasa et al.,[18] 2014	Wrong publication type
Barrett et al.,[19] 2016	Wrong publication type
Bath,[20] 2001	No full text
Baxter,[21] 2019	Wrong setting
Beghe,[22] 2000	Wrong publication type
Belfrage et al.,[23] 2009	Wrong outcomes
Bellantonio et al.,[24] 2008	Wrong patient population
Bellantonio et al.,[24] 2008	Duplicate record
Berggren et al.,[25] 2019	Duplicate record
Berggren et al.,[25] 2019	Duplicate record
Blackburn et al.,[26] 2020	Wrong study design
Bleijenberg et al.,[27] 2017	Wrong setting
Boockvar et al.,[28] 2005	Wrong study design
Bourke et al.,[29] 2019	Wrong study design
Burke et al.,[30] 2019	Wrong intervention
Cateau et al.,[31] 2021	Study protocol
Cavalieri et al.,[32] 1993	Wrong outcomes
Cebeci et al.,[33] 2008	Wrong patient population
Chan Carusone,[34] 2007	Wrong intervention
Charles et al.,[35] 2016	Wrong setting
Chen et al.,[36] 2010	Wrong intervention
Chi et al.,[37] 2004	Wrong setting
Chi et al.,[38] 2010	Wrong intervention
Choi et al.,[39] 2020	Study protocol
Clarkson et al.,[40] 2011	Wrong setting
Codde et al.,[41] 2010	Wrong outcomes
Colprim et al.,[42] 2017	Wrong language
Connolly et al.,[43] 2005	Wrong publication type
Connolly et al.,[44] 2014	Wrong outcomes
Connolly et al.,[45] 2015	Wrong outcomes

© 2022 Birtwell K et al. *JAMA Network Open*.

Connolly et al.,[46] 2016	Wrong study design
Connolly,[47] 2014	Wrong intervention
Conway et al.,[48] 2015	Wrong outcomes
Correard et al.,[49] 2020	Study protocol
Counsell,[50] 2005	Wrong setting
Craswell et al.,[51] 2020	Wrong outcomes
Crilly et al.,[52] 2005	Wrong study design
Crotty et al.,[53] 2004	Duplicate record
Crotty,[54] 2005	Duplicate record
Curtin et al.,[55] 2019	Wrong publication type
Curtin et al.,[55] 2019	Duplicate record
Curtin et al.,[55] 2019	Duplicate record
Curtin et al.,[56] 2020	Wrong intervention
Davey,[57] 2020	Ongoing study
Diaz-Gegundez et al.,[58] 2011	Wrong language
Dicks,[59] 2015	Wrong intervention
Dolansky et al.,[60] 2011	Wrong patient population
Donzé,[61] 2018	Wrong patient population
Dowling et al.,[62] 2019	Wrong publication type
Dowling et al.,[62] 2019	Duplicate record
Dutcher,[63] 2015	Wrong study design
Edmans et al.,[64] 2013	Wrong patient population
Edmans et al.,[64] 2013	Wrong patient population
Emborg et al.,[65] 2020	Wrong publication type
Epperson et al.,[66] 2020	Wrong publication type
Feast et al.,[67] 2020	Wrong outcomes
Fisher et al.,[68] 2020	Wrong patient population
Flyer et al.[69] 1988	Wrong patient population
Forbat et al.,[70] 2020	Wrong intervention
Foster et al.,[71] 2012	Ongoing study
Franks,[72] 2016	Wrong intervention
Galvin,[73] 2018	Wrong setting
Garcia-Gollarte et al.,[74] 2014	Wrong intervention
Genkin,[75] 2006	Wrong publication type
Germain et al.,[76] 1995	Wrong patient population
Gilissen et al.,[77] 2020	Study protocol
Gladman,[78] 2004	Wrong setting
Grando et al.,[79] 2009	Wrong study design
Gregersen et al.,[80] 2010	Wrong study design
Griffiths,[81] 2006	Wrong publication type
Griggs et al.,[82] 2020	Wrong publication type
Gudmundsdottir et al.,[83] 2013	Wrong publication type
Haines et al.,[84] 2020	Wrong outcomes
Heijnen et al.,[85] 2010	Wrong patient population
Heijnen,[86] 2010	Wrong outcomes
Heim et al.,[87] 2016	Wrong patient population

© 2022 Birtwell K et al. *JAMA Network Open*.

Heinrich et al.,[88] 2013	Wrong intervention
Hempenius et al.,[89] 2013	Wrong setting
Henning et al.,[90] 2017	Wrong patient population
Holdhus et al.,[91] 2018	Wrong setting
Holdhus et al.,[91] 2019	Wrong patient population
Hongsoo,[92] 2015	Study protocol
Joe,[93] 2010	Wrong language
Jones et al.,[94] 1986	Wrong setting
Kane et al.,[95] 1991	No full text
Kane et al.,[96] 2004	Wrong patient population
Kapoor et al.,[97] 2020	Wrong patient population
Karlsson et al.,[98] 2020	Wrong patient population
Kay et al.,[99] 2021	Wrong publication type
Kennelly et al.,[100] 2012	Wrong publication type
Kentish-Barnes,[101] 2019	Wrong patient population
Kerstenetzky et al.,[102] 2018	Wrong study design
Kim et al.,[103] 2016	Wrong setting
Kim et al.,[104] 2020	Wrong intervention
Kind et al.,[105] 2013	Wrong publication type
Kleinpell,[106] 2004	Wrong patient population
Koehler et al.,[107] 2009	Wrong patient population
Kotynia-English et al.,[108] 2005	Wrong intervention
Kuch,[109] 2021	Wrong intervention
Lapane et al.,[110] 2011	Wrong intervention
Lenssen et al.,[111] 2018	Wrong setting
Li et al.,[112] 2014	Wrong patient population
Lilja et al.,[113] 2000	Wrong setting
Lindegaard-Pedersen et al.,[114] 2016	Wrong patient population
Lindegaard-Pedersen et al.,[114] 2016	Wrong patient population
Lindpaintner et al.,[115] 2013	Wrong patient population
Loomer et al.,[116] 2020	Wrong setting
Malik et al.,[117] 2020	Wrong publication type
Marsden et al.,[118] 2019	Wrong study design
Marsden et al.,[118] 2020	Wrong study design
Martin et al.,[119] 2019	Wrong intervention
McDonald et al.,[120] 2018	Wrong patient population
Menon et al.,[121] 2015	Wrong publication type
Mercer et al.,[122] 2008	Wrong intervention
Mercer et al.,[123] 2015	Wrong patient population
Mestres Gonzalvo et al.,[124] 2017	Study protocol
Miller,[125] 2019	Ongoing study
Mitchell et al.,[126] 2020	Wrong outcomes
Mitchell,[127] 2013	Wrong setting
Mor et al.,[128] 2017	Study protocol
Moreau,[129] 2018	Wrong patient population
Mrak et al.,[130] 2016	Wrong intervention

© 2022 Birtwell K et al. *JAMA Network Open*.

Mueser,[131] 2015	Wrong setting
Natividad et al.,[132] 2020	Wrong publication type
Nelles,[133] 2019	Wrong publication type
Newcomer et al.,[134] 2006	Wrong patient population
Newcomer et al.,[134] 2006	Duplicate record
Nicholson,[135] 2019	Wrong publication type
Nouvenne et al.,[136] 2020	Wrong intervention
Olson et al.,[137] 1995	Wrong outcomes
O'Mahony,[138] 2018	Duplicate record
O'Reilly,[139] 2014	Wrong setting
Ouslander,[140] 2014	Duplicate record
Palmer et al.,[141] 2018	Wrong publication type
Palmer et al.,[141] 2018	Duplicate record
Pannill,[142] 2016	Wrong setting
Park et al.,[143] 2013	Wrong setting
Parker,[144] 2018	Wrong setting
Parry et al.,[145] 2003	Wrong publication type
Parry et al.,[146] 2009	Wrong patient population
Parry et al.,[146] 2009	Duplicate record
Paskulin,[147] 2016	Wrong setting
Patel et al.,[148] 2018	Wrong patient population
Pedersen,[149] 2011	Wrong setting
Pilgram et al.,[150] 2012	Wrong publication type
Piotrowski et al.,[151] 2020	Wrong intervention
Popejoy et al.,[152] 2020	Wrong setting
Provencher,[153] 2019	Wrong patient population
Ravn-Nielsen et al.,[154] 2018	Wrong publication type
Riley et al.,[155] 2020	Wrong patient population
Rojido et al.,[156] 2014	Wrong publication type
Rolland et al.,[157] 2020	Wrong study design
Ross,[158] 2008	Wrong setting
Sankaran et al.,[159] 2010	Wrong study design
Singh et al.,[160] 2021	Wrong publication type
Sorkin et al.,[161] 2016	Study protocol
Stansfield,[162] 2012	Wrong publication type
Storm et al.,[163] 2018	Wrong study design
Stranges et al.,[164] 2013	Wrong patient population
Straus et al.,[165] 2018	Wrong publication type
Sullivan,[166] 2017	Wrong intervention
Sullivan,[167] 2018	Wrong intervention
Sunner et al.,[168] 2020	Study protocol
Sunner,[169] 2019	Ongoing study
Swift,[170] 2006	Wrong publication type
Tappen et al.,[171] 2014	Wrong study design
Tappen et al.,[172] 2018	Wrong outcomes
Tappen et al.,[173] 2020	Wrong outcomes

© 2022 Birtwell K et al. *JAMA Network Open*.

Tappen,[174] 2015	Duplicate record
Tchalla,[175] 2019	Ongoing study
Testa et al.,[176] 2021	Wrong intervention
Tew Jr,[177] 2012	Wrong publication type
Toles et al.,[178] 2017	Wrong setting
Toles et al.,[179] 2018	Wrong setting
Trappes-Lomax et al.,[180] 2006	Wrong setting
Turner et al.,[181] 2001	Wrong patient population
Vangimalla et al.,[182] 2010	Wrong study design
Vogelsmeier et al.,[183] 2020	Wrong intervention
Voigt-Radloff,[184] 2018	Ongoing study
Whitehead et al.,[185] 2006	Wrong publication type
Wysocki et al.,[186] 2014	Wrong patient population
Yu et al.,[187] 2019	Wrong publication type
Zamora et al.,[188] 2011	Wrong publication type

© 2022 Birtwell K et al. *JAMA Network Open*.

eTable 3. Risk of Bias Results

The overall risk of bias judgement was classified as follows: 'low'- when the study was judged to be at low risk of bias for all domains; 'Some concerns'- when the study was judged to raise some concerns in at least one domain, but not to be at high risk of bias for any domain; 'High' – when the study was judged to be at high risk of bias in at least one domain or to have some concerns for multiple domains in a way that substantially lowered confidence in the result[193].

	Design	Risk of Bias tool	Sequence generation	Allocation concealment	Blinding a)	Blinding b)	Blinding c)	Intention-to-treat	Attritional	Selection reporting bias	Other sources of bias
Cordato et al.[194] 2018	RCT	Cochrane RoB 2.0	Low	Unclear	Low	Low	Unclear	High	Low	Low	Low
Crilly et al.[195] 2011	Non-RCT	ROBINS-I	High	Unclear	High	Unclear	Unclear	High	Unclear	Unclear	Low
Crotty et al.[53] 2004	RCT	Cochrane RoB 2.0	Low	Low	Unclear	Low	Unclear	Low	High	Low	Low
Crotty et al.[54] 2005	RCT	Cochrane RoB 2.0	Low	Low	Unclear	Low	Unclear	Low	Low	Low	Low
Elliott et al.[196] 2012 (pre-post study)	Prospective Pre-Post	ROBINS-I	Unclear	Unclear	Unclear	High	Unclear	Unclear	Low	Low	High
Harvey et al.[197] 2014	RCT	Cochrane RoB 2.0	Low	Low	Unclear	Unclear	Unclear	Low	High	Low	Low
Hullick et al.[198] 2016 (pre-post study)	Controlled Prepost design	ROBINS-I	Unclear	Unclear	Unclear	High	Unclear	Unclear	Unclear	Low	Low
Kane et al.[199] 2017	Cluster RCT	Cochrane RoB 2.0	Low	Unclear	Unclear	Unclear	Unclear	Low	Low	Low	Low
Layton[200] 2020	Quasi experimental two group design	ROBINS-I	Unclear	Unclear	Low	Unclear	Unclear	High	High	Low	High
Lee et al.[201] 2002	Matched Randomised case-control	ROBINS-I	High	Unclear	Unclear	Unclear	Unclear	High	Low	Low	Low
Mudge et al.[202] 2012	Controlled Trial	ROBINS-I	High	Unclear	Unclear	Unclear	Unclear	High	Unclear	Low	High

© 2022 Birtwell K et al. *JAMA Network Open*.

Mukamel et al.[203] 2016	RCT	Cochrane RoB 2.0	Unclear	Unclear	Unclear	Unclear	Unclear	Low	Low	Low	Low
Pedersen et al.[204] 2018	Quasi randomised	ROBINS-I	Low	High	Unclear	Unclear	Unclear	Low	Low	Low	Low
Shrapnel et al.[205] 2019	Pre/Post study	ROBINS-I	Unclear	Unclear	Unclear	Unclear	Unclear	Unclear	Unclear	Low	Low
Street et al.[206] 2015	Pre/Post study	ROBINS-I	Unclear	Unclear	Unclear	Unclear	High	Unclear	Unclear	Low	Low
**Totals n, %:**											
**High**			**3 (20%)**	**1 (7%)**	**1 (7%)**	**2 (13%)**	**1 (7%)**	**5 (33%)**	**3 (20%)**	**0 (0%)**	**3 (20%)**
**Low**			**6 (40%)**	**3 (20%)**	**2 (13%)**	**3 (20%)**	**0 (0%)**	**6 (40%)**	**7 (47%)**	**14 (93%)**	**12 (80%)**
**Unclear**			**6 (40%)**	**11 (73%)**	**12 (80%)**	**10 (67%)**	**14 (93%)**	**4 (27%)**	**5 (33%)**	**1 (7%)**	**0 (0%)**

© 2022 Birtwell K et al. *JAMA Network Open*.

eTable 4. CASP Scores for Qualitative Studies and Process Evaluations

	Study			
CASP Item [All Items Scored Yes (Y), No (N) or can't tell (CT)]	**Taylor et al.** (2007)[207] **QUAL** (NB - Conference Slides)	**Stokoe et al.** (2016)[208] **QUAL**	**Crilly et al.** (2012)[209] **PROCESS**	**Tappen et al.** (2017)[210] **PROCESS** (Note this was a questionnaire – hybrid methods)
1	Y	Y	Y	Y
2	Y	Y	Y	Y
3	Y	Y	Y	Y
4	CT	CT	Y	N
5	CT	CT	Y	Y
6	CT	Y	N	CT
7	Y	Y	Y	Y
8	CT	N	Y	Y
9	Y	Y	Y	Y
10	Y	Y	Y	Y
Total CASP items met:	6	7	9	8

Based on Kanavaki et al. 2016,[211] studies that meet 5-7 items can be considered medium quality, and studies that meet 8 or more items can be considered high quality.

eFigure 1. Hospital and ED Length of Stay

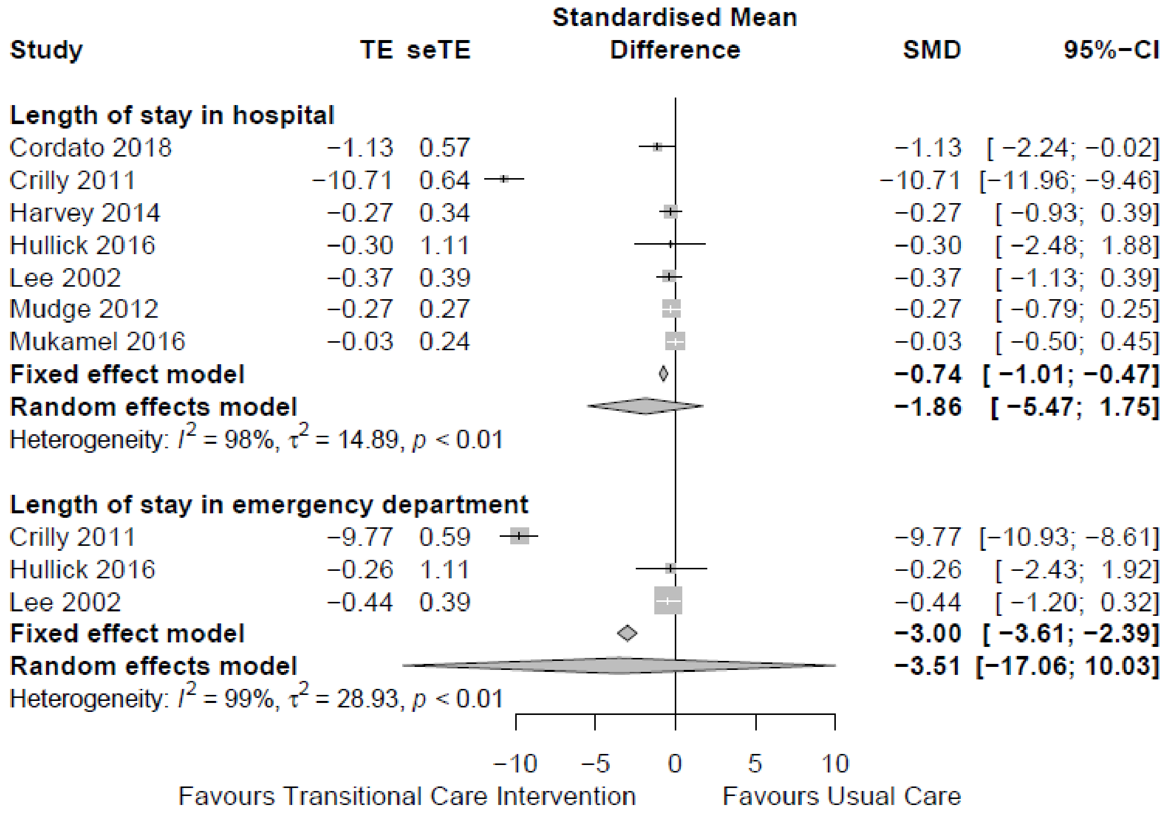

Footnote: TE=log odds ratio; seTE=standard error of the log odds ratio

© 2022 Birtwell K et al. *JAMA Network Open*.

eFigure 2. Quality of Life

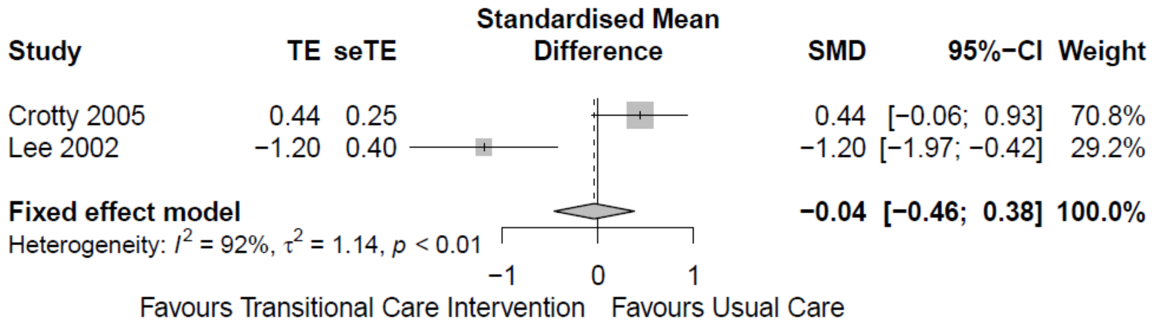

eFigure 3. Barthel Score

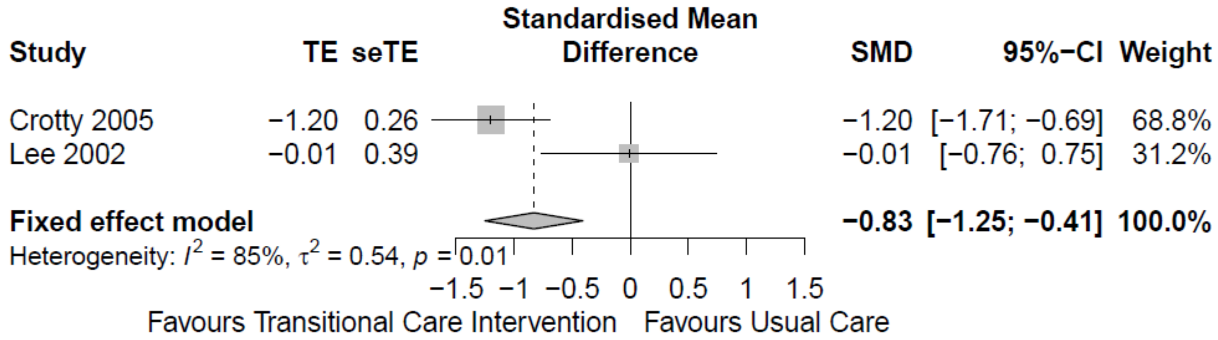

eFigure 4. Mortality Risk Ratio

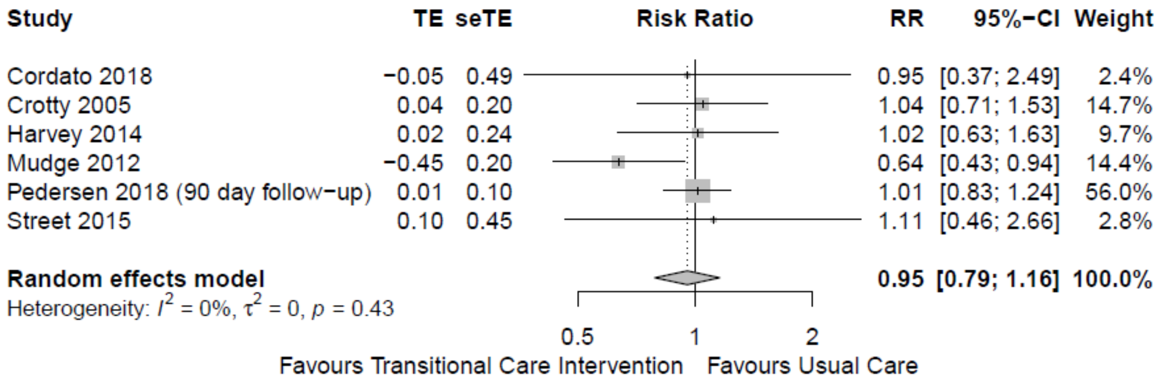

© 2022 Birtwell K et al. *JAMA Network Open*.

eFigure 5. Mortality Risk Difference

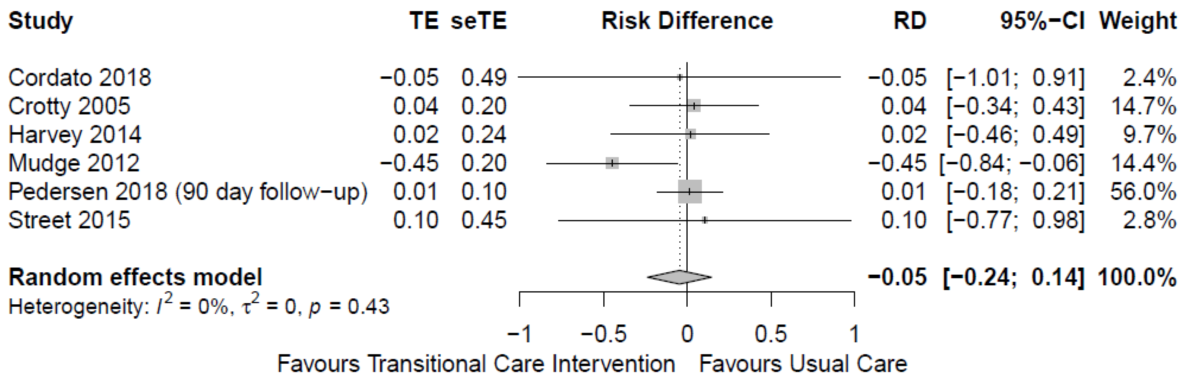

eFigure 6. Subgroup Analysis of Hospital vs ED Readmissions

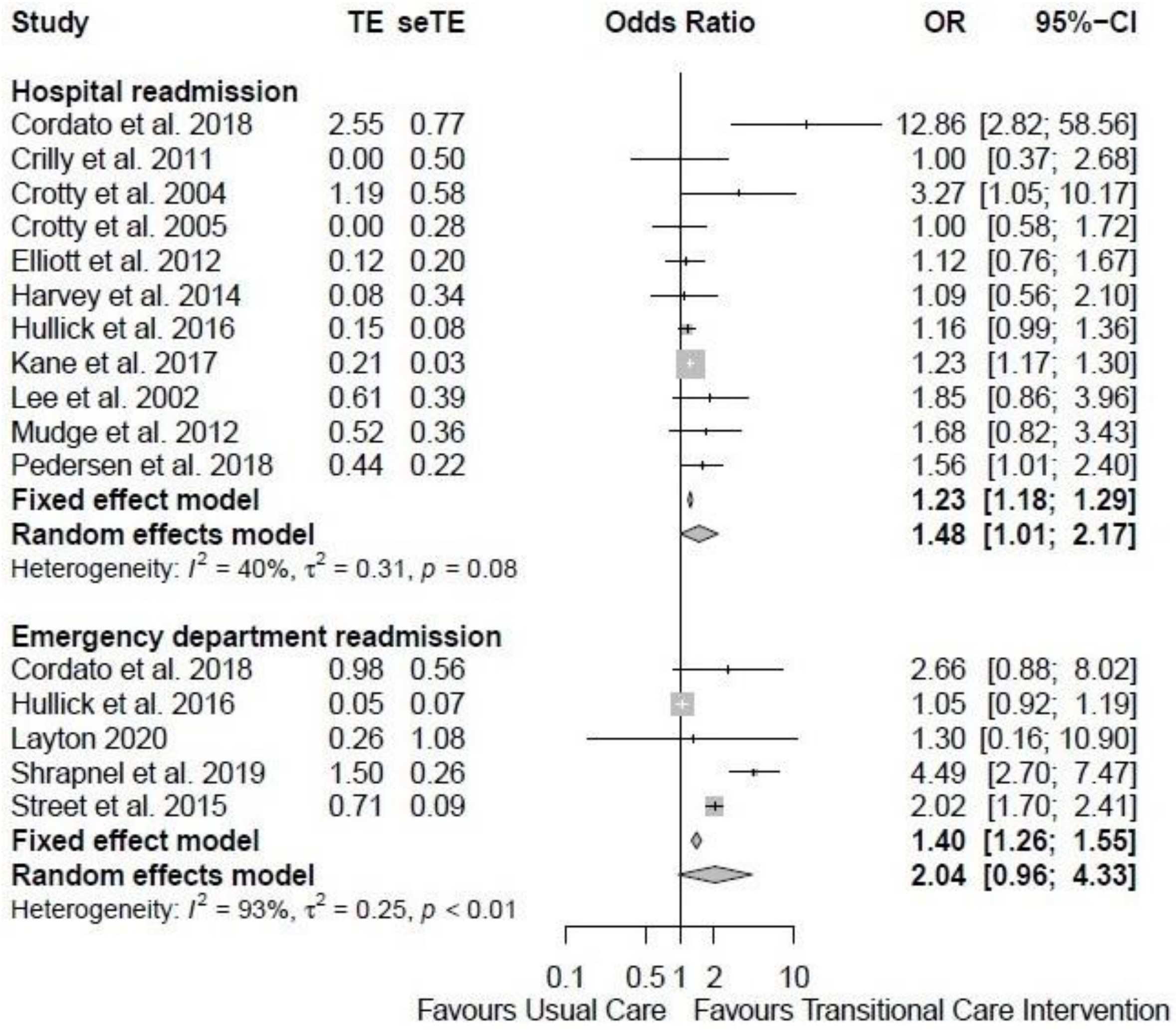

© 2022 Birtwell K et al. *JAMA Network Open*.

eFigure 7. Subgroup Analysis of Readmission by Involvement of Primary Care

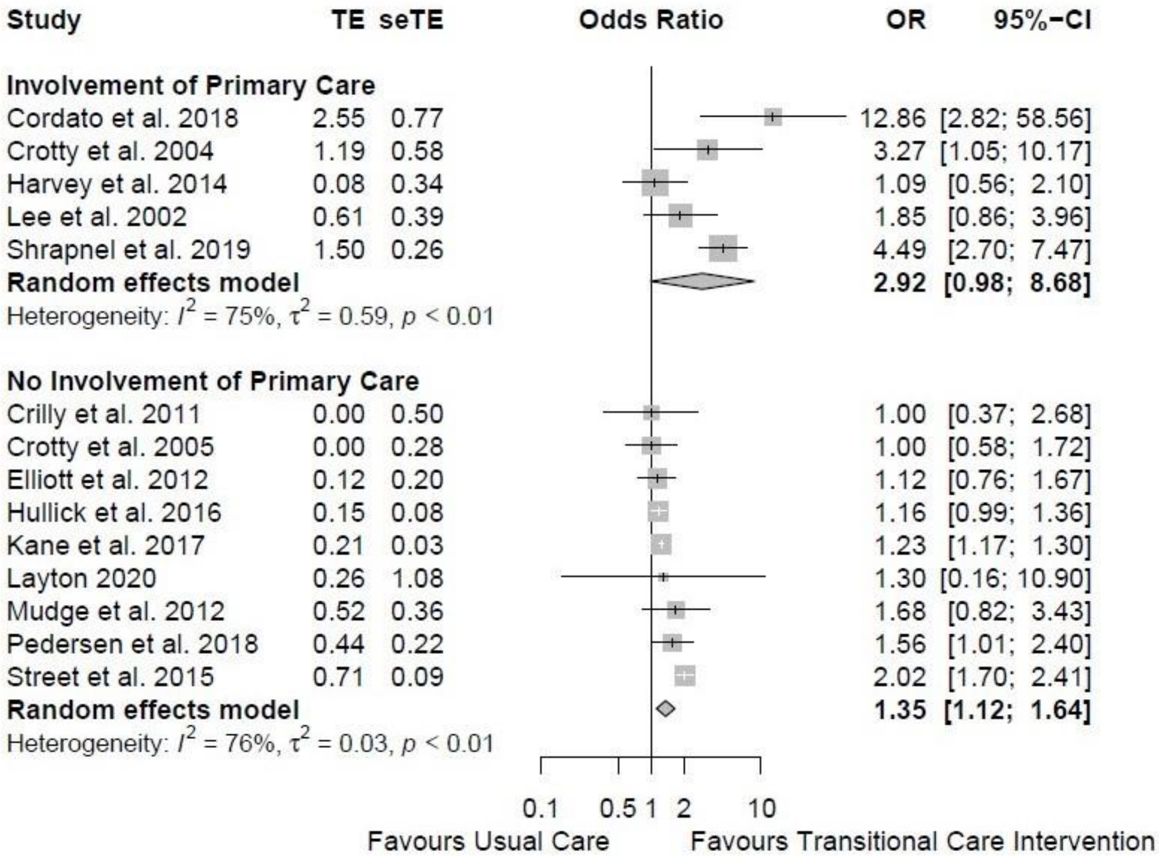

© 2022 Birtwell K et al. *JAMA Network Open*.

eFigure 8. Subgroup Analysis by Focus of Intervention

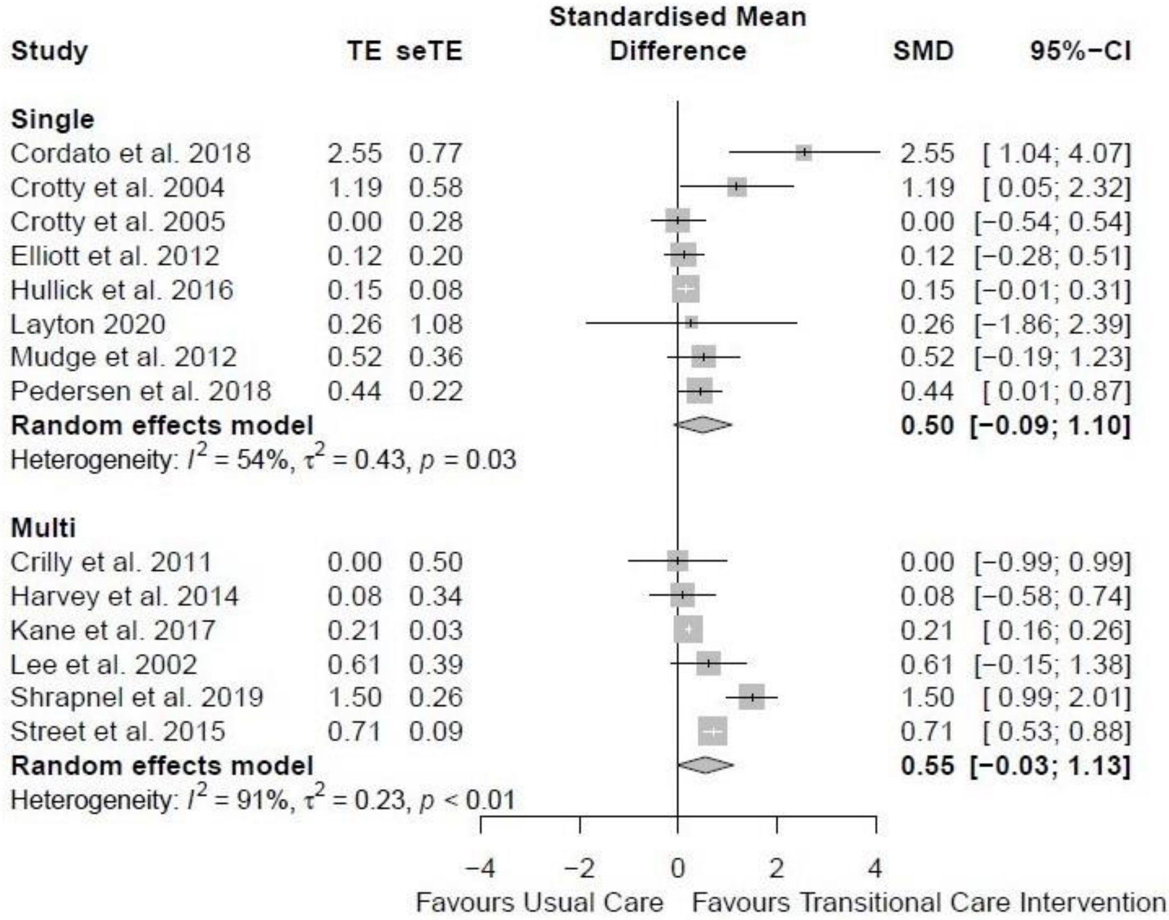

© 2022 Birtwell K et al. *JAMA Network Open*.

eFigure 9. Subgroup Analysis by Country

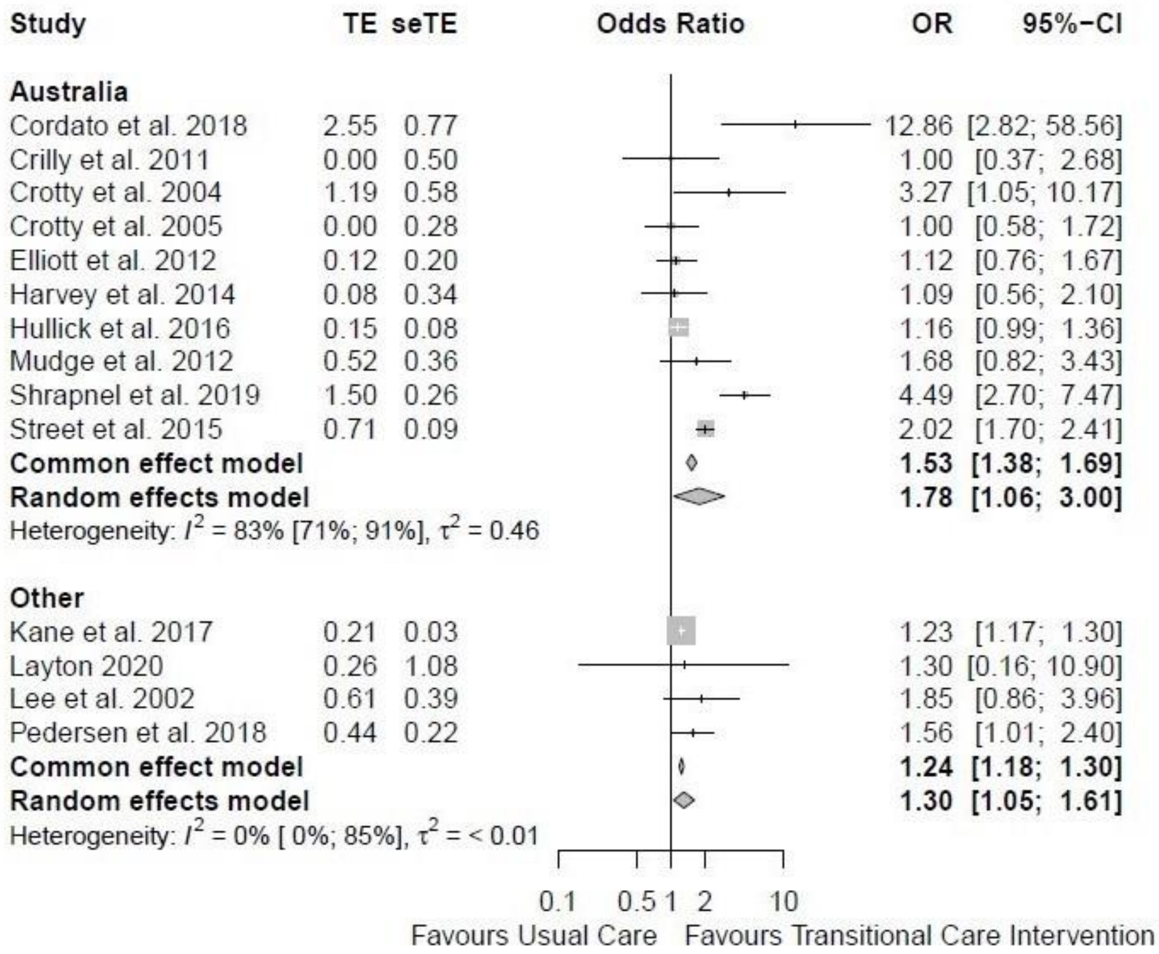

© 2022 Birtwell K et al. *JAMA Network Open*.

eFigure 10. Sensitivity Analysis: Readmission by Risk of Bias

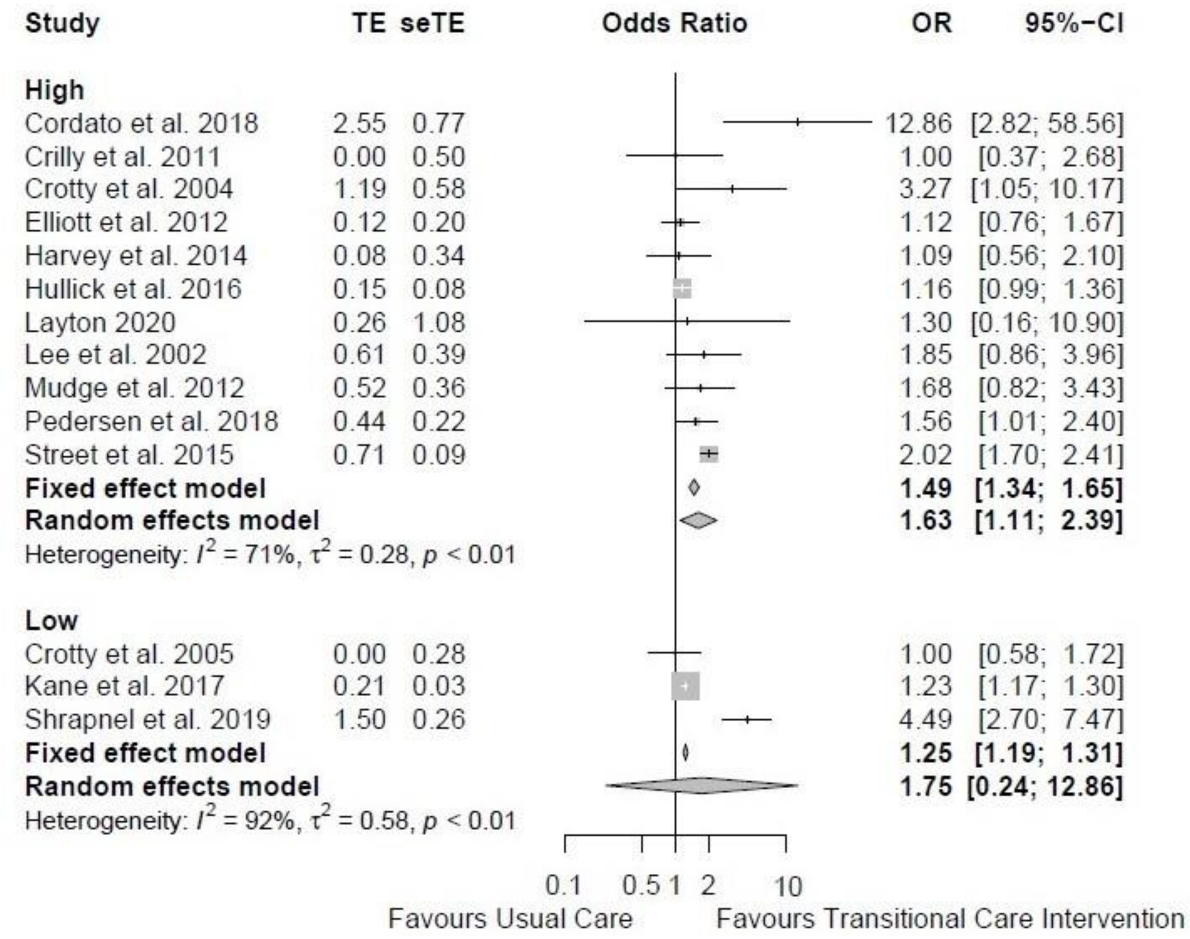

© 2022 Birtwell K et al. *JAMA Network Open*.

eReferences

1. Booth A, Harris J, Croot E, Springett J, Campbell F, Wilkins E. Towards a methodology for cluster searching to provide conceptual and contextual “richness” for systematic reviews of complex interventions: Case study (CLUSTER). *BMC Med Res Methodol*. 2013;13(1):118. doi:10.1186/1471-2288-13-118
2. Alldred DP, Kennedy M, Hughes C, Chen TF, Miller P, Cochrane Effective Practice and Organisation of Care Group. Interventions to optimise prescribing for older people in care homes. *Cochrane Database Syst Rev*. 2016;2016(2). doi:10.1002/14651858.CD009095.PUB3
3. Campbell F, Biggs K, Aldiss S, et al. Transition of care for adolescents from paediatric services to adult health services. *Cochrane database Syst Rev*. 2016;4(4). doi:10.1002/14651858.CD009794.PUB2
4. Murray J, Hardicre N, Birks Y, O’Hara J, Lawton R. How older people enact care involvement during transition from hospital to home: A systematic review and model. *Health Expect*. 2019;22(5):883. doi:10.1111/HEX.12930
5. Higgins JP, Green S. *Cochrane Handbook for Systematic Reviews of Interventions Version 5.1.0 [Updated March 2011]*. The Cochrane Collaboration; 2011. www.handbook.cochrane.org.
6. Ackermann RJ, Kemle KA. The effect of a physician assistant on the hospitalization of nursing home residents. *J Am Geriatr Soc*. 1998;46(5):610-614. doi:10.1111/J.1532-5415.1998.TB01078.X
7. Adrion C, Weiss B, Paul N, et al. Enhanced Recovery after Intensive Care (ERIC): study protocol for a German stepped wedge cluster randomised controlled trial to evaluate the effectiveness of a critical care telehealth program on process quality and functional outcomes. *BMJ Open*. 2020;10(9):e036096. doi:10.1136/BMJOPEN-2019-036096
8. Aizen E, Swartzman R, Clarfield AM. Hospitalization of nursing home residents in an acute-care geriatric department: direct versus emergency room admission. *Isr Med Assoc J*. 2001;3(10):734–738. https://pubmed.ncbi.nlm.nih.gov/11692547/. Accessed January 31, 2022.
9. Aizen E, Hindawi E, Shahla H, Elyounes AA. [The effect of an intervention on rates of nursing home residents transfers to emergency departments]. *Harefuah*. 2014;153(2). https://pubmed.ncbi.nlm.nih.gov/24716424/. Accessed January 31, 2022.
10. Akinjogbin T, Akinjogbin T, Parnell JM, Duggan M, Habermann R, Bell S. Improving Heart Failure Management in Post-Acute and Long-Term Care: A Pilot Study. *J Am Med Dir Assoc*. 2020;21(3):B22. doi:10.1016/J.JAMDA.2020.01.063
11. Allen KR, Hazelett SE, Jarjoura D, et al. The After Discharge Care Management of Low Income Frail Elderly (AD-LIFE) Randomized Trial: Theoretical Framework and Study Design. *https://home.liebertpub.com/pop*. 2011;14(3):137-142. doi:10.1089/POP.2010.0016
12. Almehlisi A, Nasser SK, Sikka N, Kulie P, Pourmand A, Joseph JW. Impact of video discharge instruction on patient comprehension, retention, and compliance in emergency department. *Acad Emerg Med Off J Soc Acad Emerg Med*. 2019;26:S9-S318. doi:10.1111/ACEM.13756
13. Altfeld S, Pavle K, Rosenberg W, Shure I. Integrating Care Across Settings: The Illinois Transitional Care Consortium’s Bridge Model. *Gener J*. 2012;36(4):98-101. https://www.proquest.com/docview/1413404255. Accessed January 31, 2022.
14. Arendts G, Deans P, O’Brien K, et al. A clinical trial of nurse practitioner care in residential aged care facilities. *Arch Gerontol Geriatr*. 2018;77:129-132. doi:10.1016/J.ARCHGER.2018.05.001
15. Arendts G. Impact of nurse practitioner care in residential aged care facilities on ED transfers: A randomised trial. *Emerg Med Australas*. 2019;31(S1):3-29. doi:10.1111/1742-6723.13239
16. Armagan E, Gultekin Al M, Engindeniz Z, Tokyay R. Compliance with protocols in transferring emergency patients to a tertiary care centre. *Injury*. 2004;35(9):857-863. doi:10.1016/J.INJURY.2003.09.038
17. Bader P. *Impact of Nurse Practitioner Visits on Readmission Rates in Skilled Nursing Homes*.; 2014.
18. Barasa S, Sconyers L, Mauldin L, Jurivich D. What is the impact of the aggressive comprehensive team approach (ACTA) on the 30 day re-Hospitalization rate? A pre and post intervention study at midway nursing home. *J Am Geriatr Soc*. 2014;62:S1-S331. doi:10.1111/JGS.12870
19. Barrett S, Baqir W, Hughes J, et al. An evaluation of the impact of a multidisciplinary review of medication in care homes on hospital admissions, out-of-hours and GP visits. *Int J Pharm Pract*. 2016;24(Supplement_3):22-102. doi:10.1111/IJPP.12289
20. Bath P. Multidisciplinary team discharge planning for severe stroke patients entering nursing home care. *Natl Res Regist*. 2001;1.
21. Baxter R. A feasibility trial of the partners at care transitions (PACT) intervention. ISRCTN registry. doi:ISRCTN51154948
22. Beghe C. An advance directive programme in nursing homes reduced health services use without affecting patient satisfaction. *Evid Based Nurs*. 2000;3(4):125-125. doi:10.1136/EBN.3.4.125
23. Belfrage MK, Chiminello C, Cooper D, Douglas S. Pushing the envelope: clinical handover from the

aged-care home to the emergency department. *Med J Aust*. 2009;190(S11). doi:10.5694/J.1326-5377.2009.TB02616.X
24. Bellantonio S, Kenny AM, Fortinsky RH, et al. Efficacy of a Geriatrics Team Intervention for Residents in Dementia-Specific Assisted Living Facilities: Effect on Unanticipated Transitions. *J Am Geriatr Soc*. 2008;56(3):523-528. doi:10.1111/J.1532-5415.2007.01591.X
25. Berggren M, Karlsson Å, Lindelöf N, et al. Effects of geriatric interdisciplinary home rehabilitation on complications and readmissions after hip fracture: a randomized controlled trial. *Clin Rehabil*. 2019;33(1):64-73. doi:10.1177/0269215518791003
26. Blackburn J, Stump TE, Carnahan JL, et al. Reducing the Risk of Hospitalization for Nursing Home Residents: Effects and Facility Variation From OPTIMISTIC. *J Am Med Dir Assoc*. 2020;21(4):545-549.e1. doi:10.1016/J.JAMDA.2020.02.002
27. Bleijenberg N, Drubbel I, Neslo RE, et al. Cost-Effectiveness of a Proactive Primary Care Program for Frail Older People: A Cluster-Randomized Controlled Trial. *J Am Med Dir Assoc*. 2017;18(12):1029-1036.e3. doi:10.1016/J.JAMDA.2017.06.023
28. Boockvar KS, Fridman B. Inter-facility transfer of patient information before and after HIPAA privacy measures. *J Am Med Dir Assoc*. 2005;6(5):310-315. doi:10.1016/J.JAMDA.2005.04.004
29. Bourke A, Batta I, Huang J, Innis A. Abstracts of the 15th International Congress of the European Geriatric Medicine Society. *Eur Geriatr Med 2020 101*. 2019;10(1):1-325. doi:10.1007/S41999-019-00221-0
30. Burke RE, Canamucio A, Glorioso TJ, Barón AE, Ryskina KL. Variability in Transitional Care Outcomes Across Hospitals Discharging Veterans to Skilled Nursing Facilities. *Med Care*. 2020;58(4):301-306. doi:10.1097/MLR.0000000000001282
31. Cateau D, Ballabeni P, Mena S, Bugnon O, Niquille A. Deprescribing in nursing homes: Protocol for nested, randomised controlled hybrid trials of deprescribing interventions. *Res Soc Adm Pharm*. 2021;17(4):786-794. doi:10.1016/J.SAPHARM.2020.05.026
32. Cavalieri TA, Chopra A, Gray-Miceli D, Shreve S, Waxman H, Forman LJ. Geriatric assessment teams in nursing homes: do they work? *J Am Osteopath Assoc*. 1993;12:1269-1272. https://pubmed.ncbi.nlm.nih.gov/8307806/. Accessed February 2, 2022.
33. Cebeci F, Çelik SŞ. Discharge training and counselling increase self-care ability and reduce postdischarge problems in CABG patients. *J Clin Nurs*. 2008;17(3):412-420. doi:10.1111/J.1365-2702.2007.01952.X
34. Chan Carusone S. Improving the care of seniors with nursing home-acquired pneumonia. 2006. doi:ISBN 9780494282090
35. Charles RJ, Singal BM, Urquhart AG, Masini MA, Hallstrom BR. Data Sharing Between Providers and Quality Initiatives Eliminate Unnecessary Nursing Home Admissions. *J Arthroplasty*. 2017;32(5):1418-1425. doi:10.1016/J.ARTH.2016.11.041
36. Chen KM, Fan JT, Wang HH, Wu SJ, Li CH, Lin HS. Silver yoga exercises improved physical fitness of transitional frail elders. *Nurs Res*. 2010;59(5):364-370. doi:10.1097/NNR.0B013E3181EF37D5
37. Garland EL, Baker AK, Howard MO. Mindfulness-Oriented Recovery Enhancement Reduces Opioid Attentional Bias Among Prescription Opioid-Treated Chronic Pain Patients. *J Soc Social Work Res*. 2017;8(4).
38. Chi YC, Chuang KY, Wu SC, Huang KC, Wu CL. The assessment of a hospital-based care management model for long-term care services. *J Nurs Res*. 2004;12(4):317-326. doi:10.1097/01.JNR.0000387516.59867.21
39. Choi JY, Kim K Il, Kim H, et al. Validation of an integrated service model, Health-RESPECT, for older patients in long-term care institution using information and communication technologies: protocol of a cluster randomised controlled trial. *BMJ Open*. 2020;10(10):e038598. doi:10.1136/BMJOPEN-2020-038598
40. Clarkson P, Brand C, Hughes J, Challis D. Integrating assessments of older people: examining evidence and impact from a randomised controlled trial. *Age Ageing*. 2011;40(3):388-391. doi:10.1093/AGEING/AFR015
41. Codde J, Arendts G, Frankel J, et al. Transfers from residential aged care facilities to the emergency department are reduced through improved primary care services: an intervention study. *Australas J Ageing*. 2010;29(4):150-154. doi:10.1111/J.1741-6612.2010.00418.X
42. Colprim D, Casco M, Malumbres J, Rodríguez G, Inzitari M. Gestión de traslados potencialmente evitables a los hospitales de agudos desde unidades de rehabilitación geriátrica: aspectos críticos de un protocolo de intervención. *Rev Esp Geriatr Gerontol*. 2017;52(2):75-79. doi:10.1016/J.REGG.2015.12.007
43. Connolly MJ, Gladman JR. Transitional Care For Elderly People. *BMJ Br Med J*. 2005;331(7527):1271-1271. https://www.jstor.org/stable/25455508?refreqid=excelsior%3A51813812e621f166d2543413cd2bf9e7. Accessed February 2, 2022.

© 2022 Birtwell K et al. *JAMA Network Open*.

44. Connolly MJ, Broad JB, Boyd M, et al. CLUSTER-RANDOMISED CONTROLLED TRIAL (RCT) OF A MULTIDISCIPLINARY INTERVENTION PACKAGE FOR REDUCING DISEASE-SPECIFIC HOSPITALISATIONS FROM LONG TERM CARE (LTC). *Age Ageing*. 2014;43(suppl_2):ii19-ii19. doi:10.1093/AGEING/AFU131.4
45. Connolly MJ, Boyd M, Broad JB, et al. The Aged Residential Care Healthcare Utilization Study (ARCHUS): A Multidisciplinary, Cluster Randomized Controlled Trial Designed to Reduce Acute Avoidable Hospitalizations From Long-Term Care Facilities. *J Am Med Dir Assoc*. 2015;16(1):49-55. doi:10.1016/J.JAMDA.2014.07.008
46. Connolly MJ, Broad JB, Boyd M, et al. The 'Big Five'. Hypothesis generation: a multidisciplinary intervention package reduces disease-specific hospitalisations from long-term care: a post hoc analysis of the ARCHUS cluster-randomised controlled trial. *Age Ageing*. 2016;45(3):415-420. doi:10.1093/AGEING/AFW037
47. Connolly M. Residential Aged Care - Avoidable Hospitalizations. *Intern Med J*. 2014;44:6-12. doi:10.1111/IMJ.12430
48. Conway J, Higgins I, Hullick C, Hewitt J, Dilworth S. Nurse-led ED support for Residential Aged Care Facility staff: An evaluation study. *Int Emerg Nurs*. 2015;23(2):190-196. doi:10.1016/J.IENJ.2014.11.005
49. Correard F, Montaleytang M, Costa M, et al. Impact of medication review via tele-expertise on unplanned hospitalizations at 3 months of nursing homes patients (TEM-EHPAD): Study protocol for a randomized controlled trial. *BMC Geriatr*. 2020;20(1):1-11. doi:10.1186/S12877-020-01546-3/FIGURES/1
50. Counsell SR. GRACE: Geriatric Resources for Assessment and Care of Elders. ClinicalTrials.gov. doi:NCT00182962
51. Craswell A, Wallis M, Coates K, et al. Enhanced primary care provided by a nurse practitioner candidate to aged care facility residents: A mixed methods study. *Collegian*. 2020;27(3):281-287. doi:10.1016/J.COLEGN.2019.08.009
52. Crilly J, Chaboyer W, Wallis M. Program evaluation of the Aged Care Early Intervention Management strategy. *Australas Emerg Nurs J*. 2005;8(3):85-130. doi:10.1016/J.AENJ.2005.08.004
53. Crotty M, Rowett D, Spurling L, Giles LC, Phillips PA. Does the addition of a pharmacist transition coordinator improve evidence-based medication management and health outcomes in older adults moving from the hospital to a long-term care facility? Results of a randomized, controlled trial. *Am J Geriatr Pharmacother*. 2004;2(4):257-264. doi:10.1016/j.amjopharm.2005.01.001
54. Crotty M, Whitehead CH, Wundke R, Giles LC, Ben-Tovim D, Phillips PA. Transitional care facility for elderly people in hospital awaiting a long term care bed: Randomised controlled trial. *Br Med J*. 2005;331(7525):1110-1113. doi:10.1136/bmj.38638.441933.63
55. Curtin D, Jennings E, Daunt R, Randles M, Gallagher P, O'Mahony D. 206 Deprescribing in Frail Older People Transitioning to Long-term Care: a Randomized Controlled Trial Using STOPPFrail Criteria. *Age Ageing*. 2019;48(Supplement_3):iii17-iii65. doi:10.1093/AGEING/AFZ103.124
56. Curtin D, Jennings E, Daunt R, et al. Deprescribing in Older People Approaching End of Life: A Randomized Controlled Trial Using STOPPFrail Criteria. *J Am Geriatr Soc*. 2020;68(4):762-769. doi:10.1111/JGS.16278
57. Davey R. Integrating pharmacists in residential aged care facilities to improve quality use of medicine. Australian New Zealand Clinical Trials Registry. doi:ACTRN12620000430932
58. Díaz-Gegúndez M, Paluzie G, Sanz-Ballester C, Boada-Mejorana M, Terré-Ohme S, Ruiz-Poza D. Evaluación de un programa de intervención en residencias geriátricas para reducir la frecuentación hospitalaria. *Rev Esp Geriatr Gerontol*. 2011;46(5):261-264. doi:10.1016/J.REGG.2011.03.001
59. Dicks RS. Ramelteon Prevention of Delirium - RCT. ClinicalTrials.gov. doi:NCT02564939
60. Dolansky MA, Zullo MD, Boxer RS, Moore SM. Initial efficacy of a cardiac rehabilitation transition program: Cardiac TRUST. *J Gerontol Nurs*. 2011;37(12):36-44. doi:10.3928/00989134-20111103-01
61. Donzé J. Transition cAre inteRvention tarGeted to High-risk patiEnts To Reduce rEADmission (TARGET-READ). ClinicalTrials.gov. doi:NCT03496896
62. Dowling MJ, Molloy U, Payne C, et al. 318 Does a Teleconference-Delivered Educational Programme (ECHO) Provided to Nursing Homes Reduce Emergency Hospital Transfers? *Age Ageing*. 2019;48(Supplement_3):iii1-iii16. doi:10.1093/AGEING/AFZ102.67
63. Dutcher S. Pharmacotherapeutic management and care transitions among nursing home residents with atrial fibrillation. 2015.
64. Edmans J, Bradshaw L, Franklyn M, Gladman JRF, Conroy S. Randomised controlled trial of specialist geriatric medical assessment for patients discharged from hospital acute assessment units. *Eur Geriatr Med*. 2013;4:S138. doi:10.1016/J.EURGER.2013.07.454
65. Emborg C, Bossow T, Moller T, Olsen N. Effect of a minimal-massive intervention to elderly living in nursing homes diagnosed with pneumonia. *Dysphagia*. 2019;35(1):133-205. doi:10.1007/S00455-019-10078-X

© 2022 Birtwell K et al. *JAMA Network Open*.

66. Epperson L. Frequent flyers: stellar strategies for emergency department superutilizers. *J Investig Med*. 2020;68(1):91-308. doi:10.1136/JIM-2019-WMRC
67. Sampson EL, Feast A, Blighe A, et al. Pilot cluster randomised trial of an evidence-based intervention to reduce avoidable hospital admissions in nursing home residents (Better Health in Residents of Care Homes with Nursing—BHiRCH-NH Study). *BMJ Open*. 2020;10(12):e040732. doi:10.1136/BMJOPEN-2020-040732
68. Fisher M, Cardoza A, Gordon A, Howard M, Neighbors L, Ragan A. Enhancing Clinical Pharmacy Specialist Involvement in Transitions of Care Focusing on Ambulatory Care Sensitive Conditions within a Veterans Affairs Healthcare System. *Pharmacy*. 2020;8(1):47. doi:10.3390/PHARMACY8010047
69. Flyer B, Rubenstein L, Robbins A, Wieland G, Henry D, Cugalj N. An intervention to improve the hospital discharge summary. *J Med Educ*. 1988;63(5):407-409. https://journals.lww.com/academicmedicine/Abstract/1988/05000/An_intervention_to_improve_the_hospital_discharge.10.aspx. Accessed February 2, 2022.
70. Forbat L, Liu WM, Koerner J, et al. Reducing time in acute hospitals: A stepped-wedge randomised control trial of a specialist palliative care intervention in residential care homes. *Palliat Med*. 2020;34(5):571-579. doi:10.1177/0269216319891077
71. Foster SJ, Boyd M, Broad JB, et al. Aged Residential Care Health Utilisation Study (ARCHUS): A randomised controlled trial to reduce acute hospitalisations from residential aged care. *BMC Geriatr*. 2012;12(1):1-6. doi:10.1186/1471-2318-12-54/FIGURES/1
72. Franks S. Transitional Care to Reduce 30-day Heart Failure Readmissions Among the Long-Term Care Elderly Population. *Nurs Res*. 2016;65(2):E37-E38. doi:10.1097/NNR.0000000000000152
73. Galvin R. Impact of an Allied Health Team in the Emergency Department on Older Adults' Care. ClinicalTrials.gov. doi:NCT03739515
74. García-Gollarte F, Baleriola-Júlvez J, Ferrero-López I, Cuenllas-Díaz Á, Cruz-Jentoft AJ. An Educational Intervention on Drug Use in Nursing Homes Improves Health Outcomes Resource Utilization and Reduces Inappropriate Drug Prescription. *J Am Med Dir Assoc*. 2014;15(12):885-891. doi:10.1016/J.JAMDA.2014.04.010
75. Genkin S. Telephone Call From Acute Care Physicians to Long Term Care Physicians: Impact on Transitional Care of Elderly Patients. ClinicalTrials.gov. doi:NCT00281749
76. Germain M, Knoeffel F, Wieland D, Rubenstein LZ. A geriatric assessment and intervention team for hospital inpatients awaiting transfer to a geriatric unit: a randomized trial. *Aging (Milano)*. 1995;7(1):55-60. doi:10.1007/BF03324293
77. Gilissen J, Pivodic L, Wendrich-Van Dael A, et al. Implementing the theory-based advance care planning ACP+ programme for nursing homes: Study protocol for a cluster randomised controlled trial and process evaluation. *BMC Palliat Care*. 2020;19(1):1-18. doi:10.1186/S12904-019-0505-7/TABLES/4
78. Gladman J. An evaluation of transitional beds - residential rehabilitation facilities for hospitalised elderly people. ISRCTN registry. doi:ISRCTN27728412
79. Grando VT, Buckwalter KC, Maas ML, Brown M, Rantz MJ, Conn VS. A trial of a comprehensive nursing rehabilitation program for nursing home residents post-hospitalization. *Res Gerontol Nurs*. 2009;2(1):12-19. doi:10.3928/19404921-20090101-06
80. Gregersen M, Damsgaard EM, Zintchouk D. Multidisciplinary geriatric intervention among nursing home residents with hip fracture reduces mortality. *Eur Geriatr Med*. 2010;1:13-89. doi:10.1016/J.EURGER.2010.07.008
81. Griffiths P. Moving elderly inpatients to a transitional care facility reduced hospital stay but increased time to transfer to long term care. *Evid Based Nurs*. 2006;9(3):90. doi:10.1136/EBN.9.3.90
82. Griggs S, Savant N, Yoon H, Park T, Halbmaier S. Pih53 Pharmacists' Interventions in Transitions of Care: Evaluating the Financial and Clinical Implications for Long-Term Care. *Value Heal*. 2020;23:S161-S162. doi:10.1016/J.JVAL.2020.04.448
83. Gudmundsdottir TK, Alexandersdottir A, Gunnarsdottir AI. Drug discrepancies at the transfer of elderly patients from hospital to nursing homes. *Int J Clin Pharm 2013 355*. 2013;35(5):909-910. doi:10.1007/S11096-013-9801-0
84. Haines TP, Palmer AJ, Tierney P, Si L, Robinson AL. A new model of care and in-house general practitioners for residential aged care facilities: a stepped wedge, cluster randomised trial. *Med J Aust*. 2020;212(9):409-415. doi:10.5694/MJA2.50565
85. Heijnen RW, Evers SM, Van Der Weijden TD, Limburg M, Schols JM. The cost effectiveness of an early transition from hospital to nursing home for stroke patients: Design of a comparative study. *BMC Public Health*. 2010;10(1):1-9. doi:10.1186/1471-2458-10-279/TABLES/1
86. Heijnen R. The (cost)-effectiveness of an early admission to and assessment in the nursing home for stroke patients: a multicentre non-randomised comparative trial. ISRCTN registry. doi:ISRCTN58135104

87. Heim N, Rolden H, van Fenema EM, et al. The development, implementation and evaluation of a transitional care programme to improve outcomes of frail older patients after hospitalisation. *Age Ageing*. 2016;45(5):643-651. doi:10.1093/AGEING/AFW098
88. Heinrich S, Rapp K, Stuhldreher N, Rissmann U, Becker C, König HH. Cost-effectiveness of a multifactorial fall prevention program in nursing homes. *Osteoporos Int 2012 244*. 2012;24(4):1215-1223. doi:10.1007/S00198-012-2075-X
89. Hempenius L, Slaets JPJ, van Asselt D, de Bock GH, Wiggers T, van Leeuwen BL. Outcomes of a Geriatric Liaison Intervention to Prevent the Development of Postoperative Delirium in Frail Elderly Cancer Patients: Report on a Multicentre, Randomized, Controlled Trial. *PLoS One*. 2013;8(6):e64834. doi:10.1371/JOURNAL.PONE.0064834
90. Henning E, Snyder R, Acus S, Dadabhoy FZ, Goroncy A, Schlaudecker J. Structured interdisciplinary bedside rounds (SIBR) for skilled nursing patients reduce hospital readmissions and length of stay. *J Am Geriatr Soc*. 2017;65:S170-S171. doi:10.1111/JGS.14915
91. Holdhus H, Bøvre K, Mathiesen L, Bjelke B, Bjerknes K. Limited effect of structured medication report as the only intervention at discharge from hospital. *Eur J Hosp Pharm*. 2019;26(2):101-105. doi:10.1136/EJHPHARM-2017-001371
92. Hongsoo K. Effectiveness of a technology-enhanced, integrated care model for frail older adults in nursing homes: a stepped-wedge cluster randomized trial. ISRCTN registry. doi:ISRCTN11972147
93. Joe YS. Development and Evaluation of a Transitional Care Program for Patients Discharged from Military Hospitals. *J Korean Acad Nurs*. 2010;40(4):599-609. doi:10.4040/JKAN.2010.40.4.599
94. Jones BJ, Meiners MR. *Nursing Home Discharges: The Results of an Incentive Reimbursement Experiment*.; 1986. https://iucat.iu.edu/iusb/4125079. Accessed February 2, 2022.
95. Kane RL, Garrard J, Buchanan JL, Rosenfeld A, Skay C, McDermott S. Improving Primary Care in Nursing Homes. *J Am Geriatr Soc*. 1991;39(4):359-367. doi:10.1111/J.1532-5415.1991.TB02900.X
96. Kane RL, Homyak P, Bershadsky B, Flood S, Zhang H. Patterns of Utilization for the Minnesota Senior Health Options Program. *J Am Geriatr Soc*. 2004;52(12):2039-2044. doi:10.1111/J.1532-5415.2004.52558.X
97. Kapoor A, Bloomstone S, Javed S, et al. Reducing Hospitalizations and Emergency Department Visits in Patients With Venous Thromboembolism Using a Multicomponent Care Transition Intervention. *Inquiry*. 2020;57. doi:10.1177/0046958019900080
98. Karlsson Å, Berggren M, Olofsson B, et al. Geriatric Interdisciplinary Home Rehabilitation After Hip Fracture in People with Dementia – A Subgroup Analysis of a Randomized Controlled Trial. *Clin Interv Aging*. 2020;15:1575-1586. doi:10.2147/CIA.S250809
99. Kay SM, Unroe K, Carnahan J. Improving communication in nursing homes using plan-do-study-act cycles of an sbar training program. *J Am Geriatr Soc*. 2021;69(S1):S1-S347. doi:10.1111/JGS.17115
100. Kennelly SM, Soh J, Van Der Poel L, et al. NHACE (Nursing Home Acute Care Evaluation): A targeted intervention in nursing home patients to enhance access to appropriate acute care. *Irish J Med Sci 2012 1817*. 2012;181(7):231. doi:10.1007/S11845-012-0842-5
101. Kentish-Barnes N. Evaluating Effectiveness of a Communication Facilitator: A Randomized Trial. ClinicalTrials.gov. doi:NCT04133753
102. Kerstenetzky L, Birschbach MJ, Beach KF, Hager DR, Kennelty KA. Improving medication information transfer between hospitals, skilled-nursing facilities, and long-term-care pharmacies for hospital discharge transitions of care: A targeted needs assessment using the Intervention Mapping framework. *Res Soc Adm Pharm*. 2018;14(2):138-145. doi:10.1016/J.SAPHARM.2016.12.013
103. Kim J, Ea E, Parish AL, Levin RF. A Nurse Practitioner–Led Heart Failure Education Program for Certified Nursing Assistants in a Long-Term Care Facility. *Ann Long-Term Care*. 2016;24(5):27-34. https://www.hmpgloballearningnetwork.com/site/altc/articles/nurse-practitioner-led-heart-failure-education-program-certified-nursing-assistants-long. Accessed February 2, 2022.
104. Kim H, Jung Y Il, Kim GS, Choi H, Park YH. Effectiveness of a Technology-Enhanced Integrated Care Model for Frail Older People: A Stepped-Wedge Cluster Randomized Trial in Nursing Homes. *Gerontologist*. 2021;61(3):460-469. doi:10.1093/GERONT/GNAA090
105. Kind A, Polnaszek B, Hovanes M, Smith M. Designation of a clinician for post-hospital follow-up care and 30-day rehospitalizations in patients discharged to nursing homes and rehabilitation facilities. *J Am Geriatr Soc*. 2013;61:S16. doi:10.1111/JGS.12263
106. Kleinpell RM. Randomized trial of an intensive care unit-based early discharge planning intervention for critically ill elderly patients. *Am J Crit Care*. 2004;13(4):335-345. https://pubmed.ncbi.nlm.nih.gov/15293587/. Accessed February 2, 2022.
107. Koehler BE, Richter KM, Youngblood L, et al. Reduction of 30-day postdischarge hospital readmission or emergency department (ED) visit rates in high-risk elderly medical patients through delivery of a targeted care bundle. *J Hosp Med*. 2009;4(4):211-218. doi:10.1002/JHM.427
108. Kotynia-English R, McGowan H, Almeida OP. A randomized trial of early psychiatric intervention in residential care: impact on health outcomes. *Int psychogeriatrics*. 2005;17(3):475-485.

doi:10.1017/S1041610205001572
109. Kuch LD. Educating residents of a continuing care retirement community to make intentional care transitions. *Diss Abstr Int Sect B Sci Eng*. 2021:No Pagination Specified-No Pagination Specified.
110. Lapane KL, Hughes CM, Daiello LA, Cameron KA, Feinberg J. Effect of a Pharmacist-Led Multicomponent Intervention Focusing on the Medication Monitoring Phase to Prevent Potential Adverse Drug Events in Nursing Homes. *J Am Geriatr Soc*. 2011;59(7):1238-1245. doi:10.1111/J.1532-5415.2011.03418.X
111. Lenssen R, Schmitz K, Griesel C, et al. Comprehensive pharmaceutical care to prevent drug-related readmissions of dependent-living elderly patients: A randomized controlled trial. *BMC Geriatr*. 2018;18(1):1-9. doi:10.1186/S12877-018-0814-3/TABLES/5
112. Li JM, Cheng SZ, Cai W, et al. Transitional care for patients with chronic obstructive pulmonary disease. *Int J Nurs Sci*. 2014;1(2):157-164. doi:10.1016/J.IJNSS.2014.05.004
113. Lilja M, Nygård L, Borell L. The Transfer of Information About Geriatric Clients in the Occupational Therapy Chain of Care: An Intervention Study. *Scand J Occup Ther*. 2000;7(2):51-59. doi:10.1080/110381200750018841
114. Lindegaard-Pedersen J, Pedersen PU, Damsgaard EM. Nutritional follow-up after discharge in malnourished geriatric patients: does the follow-up method influence the number of readmissions to hospital? | Cochrane Library. *Eur Geriatr Med*. 2016;7:S13-S14. https://www.cochranelibrary.com/central/doi/10.1002/central/CN-01252666/full. Accessed February 2, 2022.
115. Lindpaintner LS, Gasser JT, Schramm MS, Cina-Tschumi B, Müller B, Beer JH. Discharge intervention pilot improves satisfaction for patients and professionals. *Eur J Intern Med*. 2013;24(8):756-762. doi:10.1016/J.EJIM.2013.08.703
116. Loomer L, Ogarek JA, Mitchell SL, et al. Impact of an Advance Care Planning Video Intervention on Care of Short-Stay Nursing Home Patients. *J Am Geriatr Soc*. 2021;69(3):735-743. doi:10.1111/JGS.16918
117. Malik IR, Ryan M, Kennelly S, Martin R. Introduction of a new initiative for nursing home residents in the acute hospital to improve patient care. *Eur Geriatr Med*. 2020;11(1):S143. doi:10.1007/S41999-020-00428-6
118. Marsden E, Taylor A, Wallis M, et al. Effect of the Geriatric Emergency Department Intervention on outcomes of care for residents of aged care facilities: A non-randomised trial. *Emerg Med Australas*. 2020;32(3):422-429. doi:10.1111/1742-6723.13415
119. Martin RS, Hayes BJ, Hutchinson A, Tacey M, Yates P, Lim WK. Introducing Goals of Patient Care in Residential Aged Care Facilities to Decrease Hospitalization: A Cluster Randomized Controlled Trial. *J Am Med Dir Assoc*. 2019;20(10):1318-1324.e2. doi:10.1016/J.JAMDA.2019.06.017
120. McDonald E, Lee T, Wu P, Downar J, Goodine C. The medsafer pilot results: electronic deprescribing for hospitalized older adults with polypharmacy. *BMJ evidence-based Med*. 2018;23:A25. doi:10.1136/BMJEBM-2018-111070.56
121. Menon SG, Brewer M, Barrows K, et al. Effect of Telemanagement in Patients Discharged to a Nursing Facility After Heart Failure (HF) Hospitalization. *J Card Fail*. 2015;21(8):S131. doi:10.1016/J.CARDFAIL.2015.06.375
122. Mercer S, Robinson S. Educating Nursing Home Nurses on Efficient Use of the Emergency Department. *J Emerg Nurs*. 2008;34(1):74-76. doi:10.1016/J.JEN.2007.05.002
123. Mercer T, Bae J, Kipnes J, Velazquez M, Thomas S, Setji N. The highest utilizers of care: Individualized care plans to coordinate care, improve healthcare service utilization, and reduce costs at an academic tertiary care center. *J Hosp Med*. 2015;10(7):419-424. doi:10.1002/JHM.2351
124. Mestres Gonzalvo C, De Wit HAJM, Van Oijen BPC, et al. Supporting clinical rules engine in the adjustment of medication (SCREAM): Protocol of a multicentre, prospective, randomised study. *BMC Geriatr*. 2017;17(1):1-8. doi:10.1186/S12877-017-0426-3/FIGURES/2
125. Miller M. Individualized therapy for elderly patients using exercise and nutrition to reduce dependence post discharge trial. Australian New Zealand Clinical Trials Registry. doi:ACTRN12619001367134
126. Mitchell SL, Volandes AE, Gutman R, et al. Advance Care Planning Video Intervention Among Long-Stay Nursing Home Residents: A Pragmatic Cluster Randomized Clinical Trial. *JAMA Intern Med*. 2020;180(8):1070-1078. doi:10.1001/JAMAINTERNMED.2020.2366
127. Mitchell E. The CIRACT (Community In-Reach And Care Transition) Clinical and Cost-effectiveness study: the CIRACT trial. ISRCTN registry. doi:ISRCTN94393315
128. Mor V, Volandes AE, Gutman R, Gatsonis C, Mitchell SL. PRagmatic trial of Video Education in Nursing homes: The design and rationale for a pragmatic cluster randomized trial in the nursing home setting. *Clin Trials*. 2017;14(2):140-151. doi:10.1177/1740774516685298
129. Moreau L. Improving the safety and continuity of medicines management at care transitions. ISRCTN registry. doi:ISRCTN66212970
130. Mrak P, Hasandic F, Krippl P, et al. Geriatric consultation project in Austrian provincial nursing homes.

Eur Geriatr Med. 2016;7(Supplement 1). https://eugms2016-abstracts.elsevierdigitaledition.com/files/assets/basic-html/page-47.html#. Accessed February 3, 2022.
131. Mueser KT. Using The HOPES Program in Transition Care. ClinicalTrials.gov. doi:NCT02420340
132. Natividad K-A, Spivak A, Moskovits N, et al. Reengineering the Discharge Management of Heart Failure Patients Discharged to Skilled Nursing Facilities Utilizing a Multidisciplinary Team Approach Reduces 30-day Readmission Rate. *J Card Fail*. 2020;26(10):S99. doi:10.1016/J.CARDFAIL.2020.09.288
133. Nelles MP. Vun nix kütt nix - patient, geriatrician and general practitioner as a multiprofessional team for intersectoral discharge management. German Clinical Trials Register. doi:DRKS00015996
134. Newcomer R, Kang T, Graham C. Outcomes in a Nursing Home Transition Case-Management Program Targeting New Admissions. *Gerontologist*. 2006;46(3):385-390. doi:10.1093/GERONT/46.3.385
135. Nicholson C, Shrapnel S, Dent E. Culture trumps everything: The (un)expected truth about building a frailty team across the continuum for a vulnerable population. *Int J Integr Care*. 2019;19(4):374. doi:10.5334/IJIC.S3374
136. Nouvenne A, Caminiti C, DIodati F, et al. Implementation of a strategy involving a multidisciplinary mobile unit team to prevent hospital admission in nursing home residents: protocol of a quasi-experimental study (MMU-1 study). *BMJ Open*. 2020;10(2):e034742. doi:10.1136/BMJOPEN-2019-034742
137. Olson BW, Adamek ME. Streamlining Discharge Planning for Patients Returning to Nursing Homes: The Electronic Transmission of Medical Records: *J Appl Gerontol*. 1995;14(2):210-224. doi:10.1177/073346489501400205
138. O'Mahony D. Discontinuation of Long-term Medications in Older People Entering Nursing Home Care (STOPPFrail). ClinicalTrials.gov. doi:NCT03501108
139. O'Reilly M. A person-centred model of residential respite care transition support: The Transition Support-Person Centred Care (TS-PCC) Program for family caregivers and care recipients with dementia. Australian New Zealand Clinical Trials Registry. doi:ACTRN12614000043639
140. Ouslander JG. Reducing Hospitalizations of Nursing Home Residents. ClinicalTrials.gov. doi:NCT02177058
141. Palmer AJ, Si L, Haines T, Tierney P, Robinson A. Cost Analysis of Changing A Model of Care and Employing General Practitioners within Residential Aged Care Facilities Based on A Prospective, Stepped-Wedge, Cluster Randomised Trial in an Australian Setting. *Value Heal*. 2018;21:S58. doi:10.1016/J.JVAL.2018.07.442
142. Pannill FC. In older hospitalized patients, adding transitional care to in-hospital geriatric assessment did not improve ADL. *Ann Intern Med*. 2016;164(12):JC63. doi:10.7326/ACPJC-2016-164-12-063
143. Park HK, Branch LG, Bulat T, Vyas BB, Roever CP. Influence of a Transitional Care Clinic on Subsequent 30-Day Hospitalizations and Emergency Department Visits in Individuals Discharged from a Skilled Nursing Facility. *J Am Geriatr Soc*. 2013;61(1):137-142. doi:10.1111/JGS.12051
144. Parker C. PROSPER: A feasibility cluster randomised controlled trial of PeRsOnaliSed care Planning for oldER people with frailty. ISRCTN registry. doi:ISRCTN12363970
145. Parry C, Coleman EA, Smith JD, Frank J, Kramer AM. The Care Transitions Intervention: A Patient-Centered Approach to Ensuring Effective Transfers Between Sites of Geriatric Care. *Home Health Care Serv Q*. 2003;22(3):1-17. doi:10.1300/J027V22N03_01
146. Parry C, Min SJ, Chugh A, Chalmers S, Coleman EA. Further Application of the Care Transitions Intervention: Results of a Randomized Controlled Trial Conducted in a Fee-For-Service Setting. *Home Health Care Serv Q*. 2009;28(2-3):84-99. doi:10.1080/01621420903155924
147. Paskulin, Lisiane MG. Nursing Home Care Intervention Post Stroke. ClinicalTrials.gov. doi:NCT02807012
148. Patel PH, Dickerson KW. Impact of the Implementation of Project Re-Engineered Discharge for Heart Failure patients at a Veterans Affairs Hospital at the Central Arkansas Veterans Healthcare System: *Hosp Pharm*. 2018;53(4):266-271. doi:10.1177/0018578717749925
149. Pedersen JL. The Effect of Nutrition Follow up After Hospital Discharge in Undernourished Elderly. ClinicalTrials.gov. doi:NCT01345032
150. Pilgram EH, Hierzer A, Bohnstingl M, Osprian I, Schippinger W. The GECO project: Impact of a geriatric consultant service on acute hospital admissions of nursing home residents. *Eur Geriatr Med*. 2012;3:S110-S111. doi:10.1016/J.EURGER.2012.07.260
151. Piotrowski A, Meyer M, Burkholder I, et al. Effect of an interprofessional care concept on the hospitalization of nursing home residents: Study protocol for a cluster-randomized controlled trial. *Trials*. 2020;21(1):1-11. doi:10.1186/S13063-020-04325-Y/TABLES/1
152. Popejoy LL, Wakefield BJ, Vogelsmeier AA, et al. Reengineering Skilled Nursing Facility Discharge: Analysis of Reengineered Discharge Implementation. *J Nurs Care Qual*. 2020;35(2):158-164. doi:10.1097/NCQ.0000000000000413
153. Provencher V. Effectiveness of a Comprehensive Patient-centered Hospital Discharge Planning

Intervention for Frail Older Adults. ClinicalTrials.gov. doi:NCT04154917
154. Ravn-Nielsen L V., Duckert ML, Lund ML, et al. The impact of an in-hospital multifaceted clinical pharmacist intervention on the risk of readmission: a randomized clinical trial. *Pharmacoepidemiol Drug Saf*. 2018;27(S2):3-521. doi:10.1002/PDS.4629
155. Riley K, Sulo S, Dabbous F, et al. Reducing Hospitalizations and Costs: A Home Health Nutrition-Focused Quality Improvement Program. *J Parenter Enter Nutr*. 2020;44(1):58-68. doi:10.1002/JPEN.1606
156. Rojido MC, Tappen R, Newman D, Engstrom G, Ouslander J. Implementation of hospital readmission reduction interventions in U.S. Nursing homes. *J Am Geriatr Soc*. 2014;62:S99. doi:10.1111/JGS.12870
157. Rolland Y, Tavassoli N, De Souto Barreto P, et al. Systematic Dementia Screening by Multidisciplinary Team Meetings in Nursing Homes for Reducing Emergency Department Transfers: The IDEM Cluster Randomized Clinical Trial. *JAMA Netw Open*. 2020;3(2):e200049-e200049. doi:10.1001/JAMANETWORKOPEN.2020.0049
158. Ross D. Health Transitions And The Aging Population: A Framework To Measure The Value Of Rapid Rehabilitation. *Electron Theses Diss*. January 2008. https://stars.library.ucf.edu/etd/3779. Accessed February 3, 2022.
159. Sankaran S, Kenealy T, Adair A, et al. A complex intervention to support ‘rest home’ care: a pilot study. *N Z Med J*. 2010;123(1308). https://journal.nzma.org.nz/journal-articles/a-complex-intervention-to-support-rest-home-care-a-pilot-study. Accessed February 3, 2022.
160. Singh V, Lee DR, Leonard SD. Multi-targeted approach to improving transfers to a community nursing facility. *J Am Geriatr Soc*. 2021;69(S1):S73. doi:10.1111/JGS.17115
161. Sorkin DH, Amin A, Weimer DL, Sharit J, Ladd H, Mukamel DB. Rationale and study protocol for the Nursing Home Compare Plus (NHCPlus) randomized controlled trial: A personalized decision aid for patients transitioning from the hospital to a skilled-nursing facility. *Contemp Clin Trials*. 2016;47:139-145. doi:10.1016/J.CCT.2015.12.018
162. Stansfield MA. THE EFFICACY OF A NURSE PRACTITIONER CONSULTATIVE PROGRAM TO SUPPORT CARE-IN-PLACE FOR THE NURSING HOME RESIDENT EXPERIENCING A CHANGE IN CONDITION. 2012.
163. Storm M, Schulz J, Aase K. Patient safety in transitional care of the elderly: effects of a quasi-experimental interorganisational educational intervention. *BMJ Open*. 2018;8(1):e017852. doi:10.1136/BMJOPEN-2017-017852
164. Stranges P, Remington T. A multidisciplinary intervention for reducing readmissions among older adults in a patient-centered medical home. *Pharmacother J Hum Pharmacol Drug Ther*. 2013;33(10):e182-e332. doi:10.1002/PHAR.1356
165. Straus SE, Dolovich L. In hospitalized adults with polypharmacy, a multifaceted pharmacist intervention reduced readmissions at 180 days. *Ann Intern Med*. 2018;168(10):JC59. doi:10.7326/ACPJC-2018-168-10-059
166. Sullivan GJ. EFFECTS OF A STORY-SHARING INTERVENTION ON DEPRESSION AND WELL-BEING IN OLDER ADULTS TRANSITIONING TO LONG-TERM CARE. 2017.
167. Sullivan G. Effects of a Story-sharing Intervention on Depression and Well-being in Older Adults Transitioning to Long-term Care. *J Am Med Dir Assoc*. 2018;19(3):B25-B26. doi:10.1016/J.JAMDA.2017.12.078
168. Sunner C, Giles MT, Parker V, et al. PACE-IT study protocol: A stepped wedge cluster randomised controlled trial evaluating the implementation of telehealth visual assessment in emergency care for people living in residential aged-care facilities. *BMC Health Serv Res*. 2020;20(1):1-10. doi:10.1186/S12913-020-05539-1/TABLES/1
169. Sunner C. Partnerships in Aged-Care Emergency services using Interactive Telehealth (PACE-IT) incorporating telehealth visual assessment, information sharing and decision making for people living in residential aged-care facilities (RACF). Australian New Zealand Clinical Trials Registry. doi:ACTRN12619001692123
170. Swift CG. The role of medical assessment and intervention in the prevention of falls. *Age Ageing*. 2006;35 Suppl 2(SUPPL.2). doi:10.1093/AGEING/AFL083
171. Tappen R, Engstrom G, Ouslander J. Impact of interact implementation on nursing home resident safety. *J Am Geriatr Soc*. 2014;62:S143. doi:10.1111/JGS.12870
172. Tappen RM, Newman D, Huckfeldt P, et al. Evaluation of Nursing Facility Resident Safety During Implementation of the INTERACT Quality Improvement Program. *J Am Med Dir Assoc*. 2018;19(10):907-913.e1. doi:10.1016/J.JAMDA.2018.06.017
173. Tappen RM, Worch SM, Newman DO, Hain D. Evaluation of a Novel Decision Guide “Go to the Hospital or Stay Here?” for Nursing Home Residents and Families: A Randomized Trial | Research in Gerontological Nursing. *Res Gerontol Nurs*. 2020;13(6):309-319. https://journals.healio.com/doi/10.3928/19404921-20201002-01. Accessed February 3, 2022.
174. Tappen RM. Involving Nursing Home Residents and Their Families in Acute Care Transfer Decisions.

© 2022 Birtwell K et al. *JAMA Network Open*.

ClinicalTrials.gov. doi:NCT02568475
175. Tchalla A. Telemedicine - Evaluation of the Impact of a Telemedicine Device (DTM) on the Prevention of Emergency Department Visits and Hospitalizations of Nursing Home Residents Aged Polypathological. ClinicalTrials.gov. doi:NCT04008472
176. Testa L, Hardy JE, Jepson T, Braithwaite J, Mitchell RJ. Health service utilisation and health outcomes of residential aged care residents referred to a hospital avoidance program: A multi-site retrospective quasi-experimental study. *Australas J Ageing*. 2021;40(3):e244-e253. doi:10.1111/AJAG.12906
177. Tew Jr JD. Care Transitions and the Dementia Patient on JSTOR. *Generations*. 2012;36(4):109-112. https://www.jstor.org/stable/26555963. Accessed February 3, 2022.
178. Toles M, Colon-Emeric M, Naylor M, Asafu-Adjei J, Hanson L. Pilot-testing connect-home: Transitional care in skilled nursing facilities. *J Am Geriatr Soc*. 2017;65(Supplement 1):S12. doi:10.1111/JGS.14915
179. Toles M, Leeman J. Sustaining transitional care in skilled nursing facilities: A pilot study. *J Am Geriatr Soc*. 2018;66(Supplement 2):S41-S42. doi:10.1111/JGS.15376
180. Trappes-Lomax T, Ma B, Certificate Mphil P, et al. Buying Time I: a prospective, controlled trial of a joint health/social care residential rehabilitation unit for older people on discharge from hospital. *Health Soc Care Community*. 2006;14(1):49-62. doi:10.1111/J.1365-2524.2005.00596.X
181. Turner JT, Lee V, Fletcher K, Hudson K, Barton D. Measuring quality of care with an inpatient elderly population. The geriatric resource nurse model. *J Gerontol Nurs*. 2001;27(3):8-18. doi:10.3928/0098-9134-20010301-04
182. Vangimalla V, Wilson J, Paniagua M, Armbrecht E, Dalawari P. The effect of patient transfer forms on essential information from nursing home to emergency department. *J Am Geriatr Soc*. 2010;58:S206. doi:10.1111/J.1532-5415.2010.02850.X
183. Vogelsmeier A, Popejoy L, Kist S, et al. Reducing Avoidable Hospitalizations for Nursing Home Residents: Role of the Missouri Quality Initiative Intervention Support Team. *J Nurs Care Qual*. 2020;35(1):1-5. doi:10.1097/NCQ.0000000000000444
184. Voigt-Radloff S. Local, collaborative, stepped and personalised care management for older people with Chronic diseases - the randomised LoChro trial. German Clinical Trials Register. doi:DRKS00013904
185. Whitehead C, Crotty M, Wundke R, Giles LC, Tovim DB, Phillips P. A transitional care facility for hospitalised older adults awaiting a nursing home bed: a randomised controlled trial. *Intern Med J*. 2006;36(Supplement 1):A56. doi:10.1111/J.1445-5994.2006.01114.X
186. Wysocki A, Kane RL, Dowd B, Golberstein E, Lum T, Shippee T. Hospitalization of elderly Medicaid long-term care users who transition from nursing homes. *J Am Geriatr Soc*. 2014;62(1):71-78. doi:10.1111/JGS.12614
187. Yu C, Palfreyman S. Reducing hospital admissions for LRTI in an aging population. *Respirology*. 2019;24(S1):193. doi:10.1111/RESP.13492
188. Zamora Z, McCall B, LaMantia M, et al. Challenges to the implementation of a web based system to improve the transitional care of older adults. *J Am Geriatr Soc*. 2011;59(Supplement 1):S192. doi:10.1111/J.1532-5415.2011.03416.X
189. UK Public Involvement Standards Development Partnership. UK Standards for Public Involvement: better public involvement for better health and social care research. https://sites.google.com/nihr.ac.uk/pi-standards/standards. Published 2019. Accessed September 8, 2021.
190. Cochrane Consumer Network. The Statement of Principles for Consumer Involvement in Cochrane. https://consumers.cochrane.org/news/statement-principles-consumer-involvement-cochrane. Published 2017. Accessed March 8, 2021.
191. Boote J, Baird W, Sutton A. Public involvement in the systematic review process in health and social care: A narrative review of case examples. *Health Policy (New York)*. 2011;102(2-3):105-116. doi:10.1016/j.healthpol.2011.05.002
192. Braye S, Preston-Shoot M. Emerging from out of the shadows? Service user and carer involvement in systematic reviews. *Evid Policy A J Res Debate Pract*. 2005;1(2):173-194. doi:10.1332/1744264053730743
193. Sterne JAC, Savović J, Page MJ, et al. RoB 2: a revised tool for assessing risk of bias in randomised trials. *BMJ*. 2019;366. doi:10.1136/BMJ.L4898
194. Cordato NJ, Kearns M, Smerdely P, Seeher KM, Gardiner MD, Brodaty H. Management of Nursing Home Residents Following Acute Hospitalization: Efficacy of the “Regular Early Assessment Post-Discharge (REAP)” Intervention. *J Am Med Dir Assoc*. 2018;19(3):276.e11-276.e19. doi:10.1016/j.jamda.2017.12.008
195. Crilly J, Chaboyer W, Wallis M, Thalib L, Polit D. An outcomes evaluation of an Australian Hospital in the Nursing Home admission avoidance programme. *J Clin Nurs*. 2011;20(7-8):1178-1187. doi:10.1111/j.1365-2702.2010.03371.x
196. Elliott RA, Tran T, Taylor SE, et al. Impact of a pharmacist-prepared interim residential care medication

administration chart on gaps in continuity of medication management after discharge from hospital to residential care: A prospective pre- and post-intervention study (MedGap Study). *BMJ Open*. 2012;2(3). doi:10.1136/bmjopen-2012-000918
197. Harvey P, Storer M, Berlowitz DJ, Jackson B, Hutchinson A, Lim WK. Feasibility and impact of a post-discharge geriatric evaluation and management service for patients from residential care: The Residential Care Intervention Program in the Elderly (RECIPE). *BMC Geriatr*. 2014;14(1). doi:10.1186/1471-2318-14-48
198. Hullick C, Conway J, Higgins I, et al. Emergency department transfers and hospital admissions from residential aged care facilities: A controlled pre-post design study. *BMC Geriatr*. 2016;16(1):1-10. doi:10.1186/s12877-016-0279-1
199. Kane RL, Huckfeldt P, Tappen R, et al. Effects of an intervention to reduce hospitalizations from nursing homes: A randomized implementation trial of the INTERACT program. *JAMA Intern Med*. 2017;177(9):1257-1264. doi:10.1001/jamainternmed.2017.2657
200. Layton PG. Minimizing congestive heart failure readmissions from the nursing home through focused nursing education. *Diss Abstr Int Sect A Humanit Soc Sci*. 2020;81(4-A). https://hdl.handle.net/1969.6/87896.
201. Lee DTF, Lee IFK, Mackenzie AE, Ho RNL. Effects of a care protocol on care outcomes in older nursing home patients with chronic obstructive pulmonary disease. *J Am Geriatr Soc*. 2002;50(5):870-876. doi:10.1046/j.1532-5415.2002.50213.x
202. Mudge AM, Denaro CP, O'rourke P. Improving hospital outcomes in patients admitted from residential aged care: Results from a controlled trial. *Age Ageing*. 2012;41(5):670-673. doi:10.1093/ageing/afs045
203. Mukamel DB, Amin A, Weimer DL, et al. Personalizing Nursing Home Compare and the Discharge from Hospitals to Nursing Homes. *Health Serv Res*. 2016;51(6):2076-2094. doi:10.1111/1475-6773.12588
204. Pedersen LH, Gregersen M, Barat I, Damsgaard EM. Early geriatric follow-up visits to nursing home residents reduce the number of readmissions: a quasi-randomised controlled trial. *Eur Geriatr Med*. 2018;9(3):329-337. doi:10.1007/s41999-018-0045-3
205. Shrapnel S, Dent E, Nicholson C. A nurse-led model of care within an emergency department reduces representation rates for frail aged care residents. *Aging Clin Exp Res*. 2019;31(11):1695-1698. doi:10.1007/s40520-018-1101-z
206. Street M, Considine J, Livingston P, Ottmann G, Kent B. In-reach nursing services improve older patient outcomes and access to emergency care. *Australas J Ageing*. 2015;34(2):115-120. doi:10.1111/ajag.12137
207. Taylor S, Tran T, Chu M, et al. Complexities of patient transfer from hospital to aged care homes: far from achieving a smooth continuum of care. In: *Proceedings of the 28th Federal Conference of the Society of Hospital Pharmacists of Australia*. ; 2007:63.
208. Stokoe A, Hullick C, Higgins I, Hewitt J, Armitage D, O'Dea I. Caring for acutely unwell older residents in residential aged-care facilities: Perspectives of staff and general practitioners. *Australas J Ageing*. 2016;35(2):127-132. doi:10.1111/AJAG.12221
209. Crilly J, Chaboyer W, Wallis M. A structure and process evaluation of an Australian hospital admission avoidance programme for aged care facility residents. *J Adv Nurs*. 2012;68(2):322-334. doi:10.1111/J.1365-2648.2011.05740.X
210. Tappen R, Wolf D, Rahemi Z, et al. Barriers and Facilitators to Implementing a Change Initiative in Long-Term Care Using the INTERACT® Quality Improvement Program. *Health Care Manag (Frederick)*. 2017;36(3):219-230. doi:10.1097/HCM.0000000000000168
211. Kanavaki AM, Rushton A, Klocke R, Abhishek A, Duda JL. Barriers and facilitators to physical activity in people with hip or knee osteoarthritis: protocol for a systematic review of qualitative evidence. *BMJ Open*. 2016;6(11):e012049. doi:10.1136/BMJOPEN-2016-012049

© 2022 Birtwell K et al. *JAMA Network Open*.
